# Vinyl Acetate‐Enhanced Polyvinyl Chloride Gel with High Electroadhesion and Self‐Heating‐Tunability for Soft Robots in Freezing Environments

**DOI:** 10.1002/advs.202507757

**Published:** 2025-08-11

**Authors:** Chang Wei, Junshi Zhang, Lei Liu, Han Yan, Kaijun Wang, Yuzheng He, Minchao Cui, Zicai Zhu, Jihong Zhu, Weihong Zhang, Zuankai Wang, Jian Lu

**Affiliations:** ^1^ School of Mechanical Engineering Northwestern Polytechnical University Xi'an 710072 China; ^2^ Department of Mechanical Engineering The Hong Kong Polytechnic University Hong Kong SAR 999077 China; ^3^ School of Mechanical Engineering Xi'an Jiaotong University Xi'an 710049 China; ^4^ Department of Mechanical Engineering City University of Hong Kong Hong Kong SAR 999077 China

**Keywords:** electroactive polymers, electro‐adhesion, freezing environments, self‐heating, self‐reconfiguration, soft robots

## Abstract

Polyvinyl chloride gel (PVCg) exhibits versatile electromechanical properties, making it highly promising for soft robots. However, conventional PVCg with excessive plasticizers generates a significant amount of heat and suffers from premature electrical breakdown during electro‐induced actuation, seriously limiting its widespread application. Here, a novel strategy is demonstrated to simultaneously regulate the heat generation and improve the electromechanical properties of PVCg by introducing polyvinyl chloride‐co‐vinyl acetate (PVCVA) to fabricate PVCVA gel (PVCVAg). Notably, the proposed PVCVAg exhibits over 50% reduction in heat generation, 15‐fold extended lifespan (from 200 s to over 3000 s), and 2.15 times higher electro‐adhesion force (from 13.8 to 29.6 kPa) compared to the state‐of‐the‐art PVCg. Based on the improved electroactive properties of PVCVAg, electro‐actuation, adhesion, and tunable heating are integrated into a soft robot to achieve fast crawling, module self‐reconfiguration within millimeter dimensions via electroadhesive connections, and on‐demand environmental thermal interaction without requiring auxiliary heaters. Moreover, these capabilities are validated through various tests, including self‐reconfiguration in maze‐like confined spaces, operation at −50 °C, and collaborative aero‐engine blisk inspection and ice melting in freezing environments. These demonstrations highlight the application potential of the integrated multifunctional PVCVAg devices in complex and extreme environments.

## Graphical abstract

This study presents a vinyl acetate‐enhanced electroactive polyvinyl chloride gel with low‐voltage deformation, strong electro‐adhesion, and controlled heating. Leveraging these synergistic properties, this work develops soft robots capable of fast locomotion, swarm reconfiguration, and low‐temperature operation (‐50°C). The robot performs collaborative aero‐engine inspection and ice melting in freezing environments, demonstrating transformative potential of this material for intelligent systems.

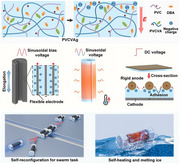

## 1 Introduction

Electroactive polymers (EAP) are a fascinating class of electrically responsive materials for soft robots.^[^ ^1^, ^2^, ^3^, ^4^, ^5^, ^6^, ^7^ ^]^ Various EAP‐based actuators have been developed, including planar, conical, balloon, multilayer stacked, and rolled configurations.^[^ ^8^, ^9^, ^10^, ^11^, ^12^ ^]^ Despite remarkable progress, current studies predominantly focus on singular electroactive responses, i.e., deformation under electric stimuli. Hence, future applications in multifunctional systems demand diverse responsive behaviors within a single EAP material, which would not only enhance functional integration and adaptability but also reduce system complexity.^[^ ^13^, ^14^, ^15^, ^16^, ^17^ ^]^

Recently, plasticized polyvinyl chloride gel (PVCg) has emerged as an electroactive material with versatile responses under a low electric field, including planar deformation with flexible electrodes and adhesion‐induced creep with rigid electrodes,^[^ ^18^, ^19^ ^]^ which opens new possibilities for soft robots. The responsiveness of PVCg is intrinsically governed by plasticizers, which not only reduce intermolecular interactions between PVC chains but also undergo electric field‐directed migration. This migration facilitates the formation of a near‐anode solvent‐rich (S‐R) layer, which arises from the enrichment of polarized plasticizer molecules combined with negative charges.^[^ ^20^, ^21^ ^]^ Notably, electroactive properties of PVCg strongly depend on the S‐R layer, and an increase in plasticizer content tends to enhance the electromechanical properties. However, an excessive amount of plasticizer results in severe heating and premature breakdown, which is attributed to significant plasticizer movement and large leakage currents.^[^ ^22^, ^23^ ^]^ Although several studies have focused on the modification of PVCg, these efforts predominantly target improvements in deformation or adhesion properties while neglecting or exacerbating issues of heat generation and breakdown strength.^[^ ^24^, ^25^, ^26^, ^27^, ^28^, ^29^, ^30^, ^31^ ^]^ For example, introducing ionic liquids or solid conductive fillers achieves enhanced electromechanical responses through increased conductivity but simultaneously causes elevated leakage currents. Therefore, there is a trade‐off between the electroactive performance and device stability. Suppressing these instabilities while ensuring significant output displacements and forces is imperative for PVCg's practical application.

Although numerous advanced soft robots have emerged over the past decade with the development of EAP,^[^ ^32^, ^33^, ^34^, ^35^, ^36^, ^37^, ^38^, ^39^, ^40^ ^]^ the aforementioned material‐level limitations mirror the challenge of multi‐functioning with the scaling down of robot dimensions, where space constraints and payload limitations become critical.^[^ ^41^ ^]^ Most miniaturized soft robots have simple locomotion capabilities or carry simple devices such as miniature cameras or lasers. Complex functions, such as self‐reconfigurability and self‐heating^[^ ^42^, ^43^, ^44^ ^]^ have yet to be realized. Self‐reconfigurable modular robots (SRMR) can change their configuration by physically interconnecting with each other,^[^ ^45^, ^46^, ^47^ ^]^ enabling diverse responses to complex environments. The connection/disconnection mechanism serves as the core component determining integral system performance, with current solutions primarily relying on mechanical, magnetic, or pneumatic principles.^[^ ^48^, ^49^, ^50^, ^51^ ^]^ Nevertheless, these conventional approaches inevitably introduce excessive system bulkiness and structural complexity, resulting in current SRMR designs that typically exceed 5 cm in dimension while weighing over 15 g (Table S1, Supporting Information). The fundamental size‐weight limitation has precluded the development of SRMR in the miniaturized systems, which could revolutionize applications ranging from confined‐space inspection to collective swarm operations. Similar problems are also encountered in the development of self‐heating robots. Controlled self‐heating is particularly important for robots interacting with the external environment.^[^ ^52^, ^53^ ^]^ Significant efforts have been devoted to the development of heating methods for soft or flexible materials/structures, including ultrasonic, magnetic, and electronic methods.^[^ ^54^, ^55^, ^56^, ^57^ ^]^ However, most soft electric heaters merely have a single function, namely heating, and integrating an independent heating component into a small‐scale robot requires an additional payload, which may hinder mobility and controllability.

**Figure 1:**
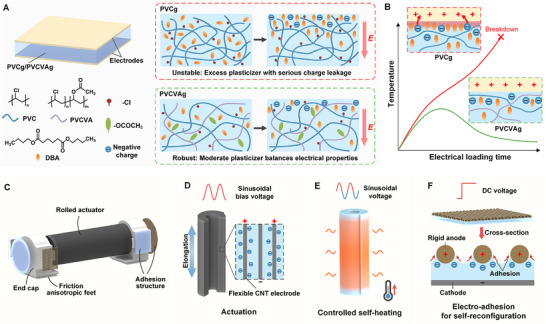
Schematic diagram of materials design and soft robots with various functions. A) Schematic illustration of chemical structures and electroactive mechanism of PVCg and PVCVAg. B) Variation of the temperature‐electrical loading time curves of PVCg and PVCVAg. C) Structural design of soft robots. D) Mechanisms of deformation for rolled multi‐layered PVCVAg actuators. E) Concept of controlled self‐heating of actuators under a sinusoidal voltage. F) Mechanisms of electro‐adhesion based on PVCVAg.

In this work, we present a PVCg‐based EAP exhibiting low‐voltage deformation, strong electro‐adhesion, and controlled heating. The uncontrollable destructive heating was suppressed and electromechanical performance was enhanced simultaneously by introducing polyvinyl chloride‐co‐vinyl acetate (PVCVA) into PVCg, resulting in a composite called PVCVA gel (PVCVAg) (**Figure** **1**A,B). The optimized rolled multi‐layered PVCVAg actuators demonstrate an over 50% reduction in heat generation and a more than 15‐fold extended lifespan compared to pristine PVCg, alongside 3.84‐ and 1.75‐fold improvements in actuation sensitivity and output force, respectively. The electro‐adhesion strength has also been significantly improved to reach ≈30 kPa under an ultra‐low electric field of merely 2 V µm^−1^, representing an order‐of‐magnitude enhancement in adhesion pressure with a tenfold reduction in required electric field compared to traditional electrostatic adhesion. Beyond material‐level heat suppression, our work also highlights on‐demand heating through a previously unexplored voltage strategy, achieving the controllable amplification or suppression of heat for PVCg‐based materials. The improvement and integration of electroactive properties can translate to soft robotic functionalities unattainable with current EAP (Figure 1C). The advances in actuation and stability of the designed rolled PVCVAg actuators (Figure 1D) enable the robot to operate at ultra‐low voltage (72.5 V), which demonstrates a 75% reduction in driving voltage relative to state‐of‐the‐art systems. The controllable self‐heating properties (Figure 1E) of PVCVAg actuators facilitate robots to achieve environmentally thermal interaction without auxiliary heaters in freezing environments and prevent ice accumulation. In addition, breakthroughs in electro‐adhesion based on PVCVAg/cathodes and rough anodes (Figure 1F) enable self‐reconfigurable robots to achieve module connection within millimeter dimensions without relying on complex microstructures. The high‐strength docking between modules can easily support weights exceeding 20 g (730× the adhesion structure's weight) and withstand dynamic shocks. The various capabilities in actuation, adhesion, swarm task execution, and self‐heating realized through PVCVAg enable our robots to operate in extreme environments and perform complex tasks.

## 2 Results and Discussion

### 2.1 Electro‐Mechanical Properties of PVCVAg

**Figure 2:**
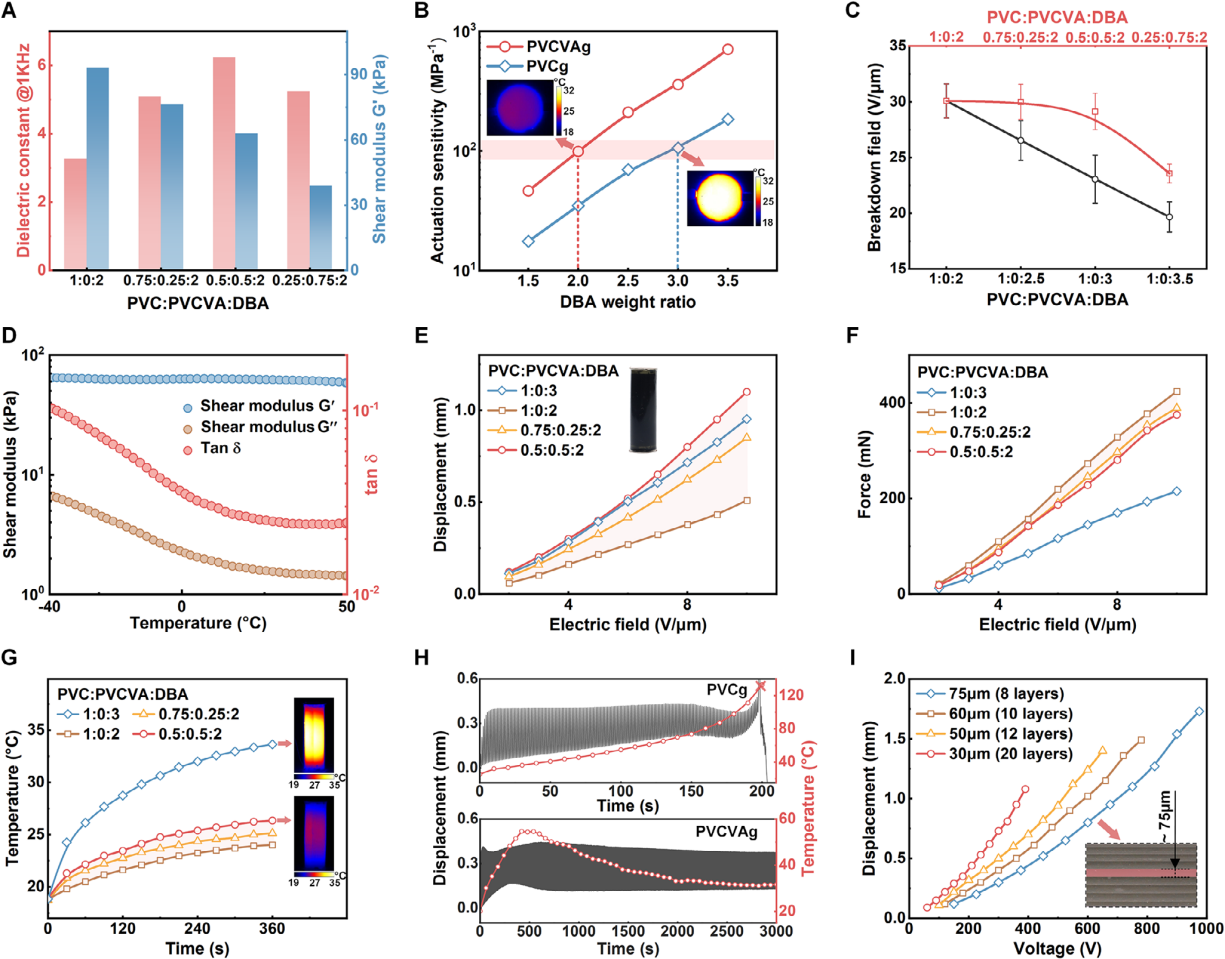
Electro‐mechanical properties of PVCVAg. A) Dielectric constant (1 kHz, 25 °C) and Shear modulus (1 Hz, 25 °C) of PVCVAg with different weight ratios of PVC and PVCVA. B) Actuation sensitivity of PVCg and PVCVAg with different weight ratios of DBA. For PVCVAg, the weight ratio of PVC and PVCVA is 0.5:0.5, and the DBA weight ratio is the DBA relative to PVC and PVCVA together. For PVCg, the DBA weight ratio is the DBA relative to PVC. The insets show the corresponding infrared images under an electrical field of 3 V µm^−1^. C) The breakdown field of PVCg and PVCVAg with different weight ratios of PVC, PVCVA, and DBA. Error bars, standard deviation of the mean for *n* = 3 samples. D) The shear modulus (G’, G”) and tan δ of PVCVAg (0.5:0.5:2), evaluated from −40 to 50 °C. E,F) Static displacement and static output force of rolled PVCVAg actuators with different weight ratios of PVC, PVCVA, and DBA. G) The temperature of rolled PVCVAg actuators with different weight ratios of PVC, PVCVA, and DBA at 160 V (DC voltage), the insets show the infrared images at the 360 s. H) The variation of displacement and temperature of PVCg (1:0:3) and PVCVAg (0.5:0.5:2) actuators (1 Hz, 375V). I) Static displacement of PVCVAg actuators with different single‐layer thicknesses, the inset shows the cross‐section of multilayered PVCVAg (8 layers with a single‐layer thickness of 75 µm).

For traditional EAP, the elastic modulus and dielectric constant play primary roles in determining the performance under an applied electric field. However, in the case of PVCg‐based materials, the dynamic migration of plasticizers (typically dibutyl adipate (DBA)) and negative charges and the evolution of near‐anode S‐R layer also largely determine their electroactive responsiveness.^[^ ^24^, ^58^, ^59^ ^]^ As a dipole plasticizer, DBA can reduce stiffness and increase the dielectric constant (Figures S1 and S2, Supporting Information), as well as facilitate the formation of S‐R layer. Nevertheless, excessive DBA significantly increases the conductivity and results in serious charge leakage in S‐R layer,^[^ ^60^, ^61^, ^62^ ^]^ which not only deteriorates responsive performance but also gives rise to unacceptable heat generation and premature breakdown, especially for compact and energy‐intensive applications. In this study, alongside DBA, we introduce PVCVA to further regulate the material's properties, resulting in PVCVAg. Materials with different PVC, PVCVA, and DBA contents are denoted as ‘x1:x2:x3’, where x1, x2, and x3 represent the weight ratios of PVC, PVCVA, and DBA, respectively. As depicted in **Figures** **2**A and S3 (Supporting Information), the dielectric constant of PVCVAg with the weight ratio of 0.5:0.5:2 (6.23 at 1 kHz) is nearly double that of PVCg with the weight ratio of 1:0:2 (3.27 at 1 kHz). The increase in the dielectric constant can be attributed to the interfacial polarization induced by the introduction of PVCVA, which contains highly polar vinyl acetate (VA) groups. Nevertheless, excessive PVCVA contents may cause a reduction in the dielectric constant of PVCVAg, resulting from the deterioration of the interfacial polarization. Moreover, the presence of VA groups disrupts the strong secondary bonding between the PVC chains, leading to a monotonical reduction in the modulus when the weight ratio changes from 1:0:2 to 0.25:0.75:2 (Figure 2A; Figure S4, Supporting Information).

The trade‐off between electro‐mechanical performance and instability (i.e., overheating and breakdown) can be alleviated by introducing PVCVA into PVCg. In Figure 2B, the actuation sensitivity,^[^ ^63^, ^64^ ^]^ defined as the ratio of dielectric constant to shear modulus, is calculated to evaluate the deformation capability. The introduction of PVCVA significantly improves the actuation sensitivity, which shows a 3.84‐fold increase in PVCVAg compared to PVCg at a DBA weight ratio of 3.5. Notably, the actuation sensitivity of PVCVAg with DBA weight ratio of 2 (0.5:0.5:2) is similar to that of PVCg with DBA weight ratio of 3 (1:0:3), which are ≈99.08 and 106.05, respectively (Figure 2B). However, under an electric field of 3 V µm^−1^, the changes in temperature due to heating of the planar actuators (Figure S5, Supporting Information) based on PVCVAg are smaller than that of PVCg, which are 5.17 and 10.65 °C (room temperature of 19 °C), respectively. Additionally, by introducing PVCVA rather than simply increasing the plasticizer content, the breakdown field can avoid severe degradation. For example, although PVCVAg with a weight ratio of 0.5:0.5:2 and PVCg with a weight ratio of 1:0:3 have similar actuation sensitivity, the breakdown field of the former is ≈25% higher than that of the latter (Figure 2C). Temperature effects on the mechanical properties of PVCVAg are also considered (Figure 2D). The shear storage modulus remains relatively stable within the temperature range of −40 to 50 °C, and the shear loss modulus decreases at low temperatures and stabilizes somewhat after 20 °C, indicating a wide temperature range of usability.

Achieving a high force output from the actuator with limited volume and weight is a key factor in determining the movement performance of soft robots. Accordingly, we roll stacked multi‐layers of PVCg or PVCVAg into compact actuators. The detailed fabrication process is shown in Note S1 and Figure S6 (Supporting Information). The doctor blade coating method is used to fabricate each layer of PVCg or PVCVAg, and the pad printing method is used to transfer carbon nanotube (CNT) electrodes with optimized material dosage (Figure S7 and Note S2, Supporting Information) before stacking the next layer of gel. The evaporation of the solvent (tetrahydrofuran, THF) can be accelerated by delicately adjusting the temperature. Consequently, it is possible to fabricate each layer of PVCg/ PVCVAg in less than an hour, instead of several days as previously reported.^[^ ^65^, ^66^ ^]^ Then, the multilayered PVCg or PVCVAg films are rolled into cylindrical actuators, as shown in the inset of Figure 2E. The fabricated rolled actuator is 20 mm in length and 7 mm in diameter.

The performance of pristine PVCg rolled actuators (1:0:3) and the impact of varying amounts of PVCVA (1:0:2, 0.75:0.25:2, and 0.5:0.5:2) are examined. For the actuator with a ratio of 1:0:3, the displacement is ≈0.95 mm at 10 V µm^−1^ (Figure 2E), and the maximum output force is 215 mN (Figure 2F). However, the electroactive response is accompanied by significant heating, ultimately reaching over 33 °C under an electric field strength of only 2.13 V µm^−1^ (Figure 2G). Reducing the amount of DBA (1:0:2) decreases the heat generation but also leads to a noticeable deterioration in electro‐mechanical performance. Introducing PVCVA into pristine PVCg can significantly improve the deformation and output force. For example, the rolled actuator with a ratio of 0.5:0.5:2 can achieve comparable deformation to that with a ratio of 1:0:3 but with a notably improved output force (375 mN at 10 V µm^−1^), which is 1.75‐fold higher than that of PVCg actuator (1:0:3). More importantly, under the same electric field strength (2.13 V µm^−1^), the heat generated in PVCVAg actuators with a ratio of 0.5:0.5:2 is reduced more than 50% compared with that in PVCg actuators with a ratio of 1:0:3, reaching only ≈25 °C. Because the higher dielectric constant of the material with a ratio of 0.5:0.5:2, the output force of PVCVAg actuators with a ratio of 0.5:0.5:2 is similar to that of the 0.75:0.25:2 PVCVAg actuator, but the displacement is significantly larger. Therefore, the material ratio used for the subsequent rolled actuators was chosen to be 0.5:0.5:2.

Furthermore, under a sinusoidal bias voltage (high level 375 V, low level 0 V, frequency 1 Hz), the long‐term time‐histories of the deformation and temperature of PVCg actuator (1:0:3) and PVCVAg actuator (0.5:0.5:2) are monitored. The temperature of PVCg actuator increases and reaches ≈70 °C after 150 s, accompanied by a decreasing deformation. After that, the temperature rises dramatically, exceeding 120 °C at 200 s and causing the actuator to break down. Although the PVCVAg actuator also shows an increase in temperature and a decrease in deformation during the initial stage, the temperature peaks at 53 °C ≈500 s and gradually reduces to ≈33 °C thereafter. In the meantime, the deformation of the PVCVAg actuator turns to be stabilized, without breakdown occurrence after a duration of 3000 s, which demonstrates a more than 15‐fold extension in lifetime. In summary, we achieved synergistic optimization of plasticizer content and polymer chains to avoid overheating and premature breakdown caused by excessive plasticizer migration, while ensuring deformation and output force performance. This makes our modified PVCg more promising for practical applications.

Moreover, reducing the single‐layer thickness in the multilayer‐based rolled actuators can significantly reduce the required driving voltage, as shown in Figure 2I and Figure S8 (Supporting Information), in which we fabricate four types of rolled PVCVAg actuators with different single‐layer thicknesses and ensure that the total thicknesses of the multilayers are the same by regulating the number of layers. Figure 2I shows the significant difference between the displacements of PVCVAg actuators with different single‐layer thicknesses. For example, ≈375 V is required to achieve 2% strain for the actuator with a single‐layer thickness of 75 µm, whereas only 200 V is required for the actuator with a single‐layer thickness of 30 µm. That is, reducing the single‐layer thickness of PVCVAg allows the actuation voltages to be further reduced if needed.

### 2.2 Movement of PVCVAg‐Based Soft Robots

**Figure 3:**
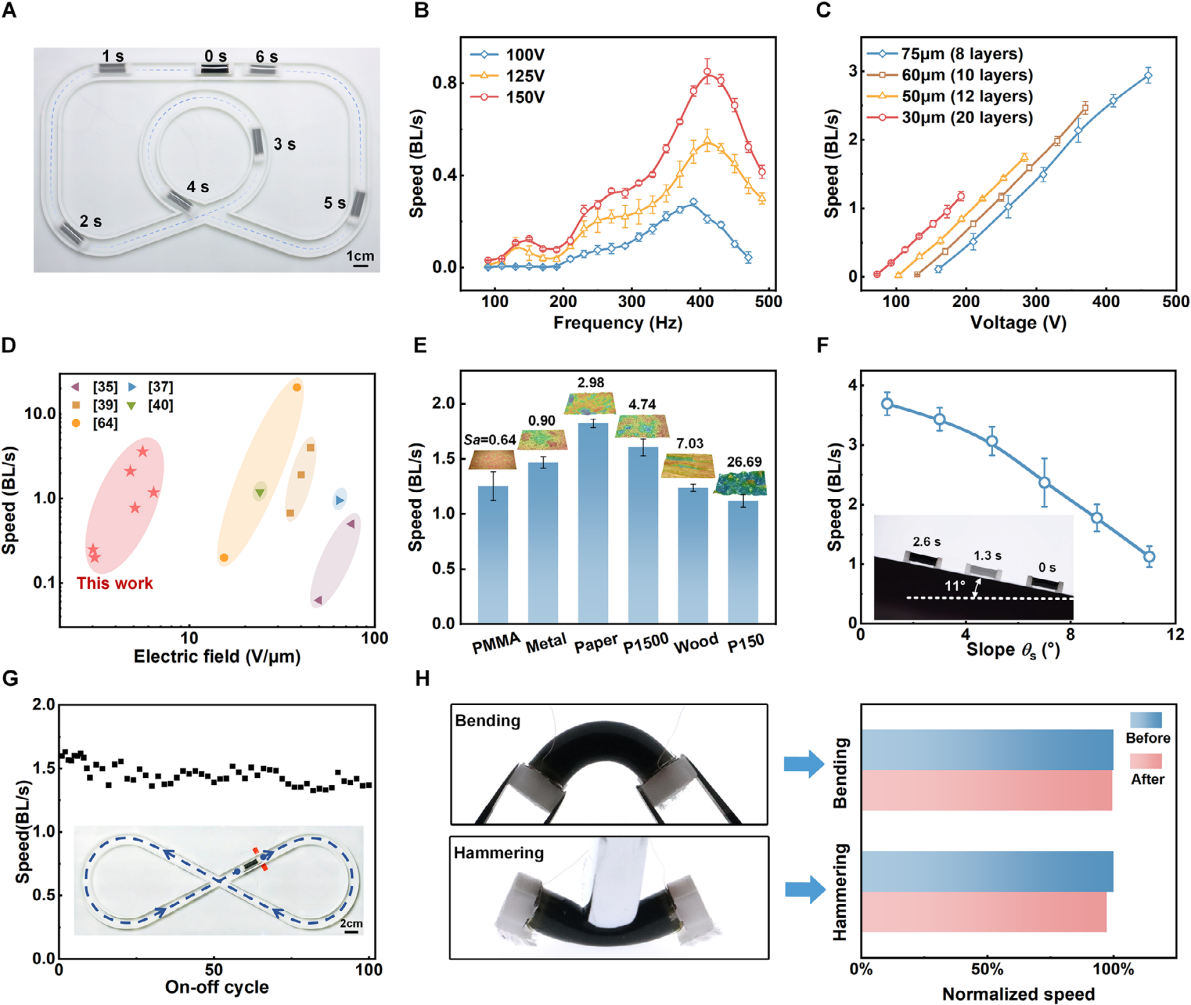
Movement of soft robots based on rolled PVCVAg actuators. A) Snapshots of the crawling PVCVAg‐based soft robot. B) The relationship between crawling speeds and actuation frequency (using a rolled PVCVAg actuator with a single‐layer thickness of 30 µm). C) Crawling speeds of soft robots with different single‐layer thicknesses of the rolled PVCVAg actuators. The frequencies are 340, 355, 360, and 370 Hz for single‐layer thicknesses of 75, 60, 50, and 30 µm, respectively. D) The driving electric field of similar EAP robots smaller than 5 cm versus crawling speed. E) The crawling speeds of soft robots on different surfaces, including Polymeric Methyl Methacrylate (PMMA), metal (aluminum), paper, wood board, sandpaper with P1500 and P150. The insets show topography of different surfaces and the values of roughness profile *Sa*. F) Crawling speeds of soft robots with different slope angles and snapshots of climbing a slope of 11°. G) The relationship between the crawling speed and the on‐off cycling of the robot. The inset shows the test path. H) Normalized crawling speed before and after mechanical shock (bending and hammering). Error bars, standard deviation of the mean for *n* = 3 samples.

Using rolled PVCVAg actuators, we fabricate soft robots (**Figure** **3**A; Movie S1, Supporting Information) by adding two friction anisotropic feet.^[^ ^39^, ^64^, ^67^ ^]^ The detailed fabrication process is presented in Methods. Friction anisotropic structures are utilized to achieve directionally‐controlled motion (Figure S9, Supporting Information). As shown in Figure 3B, there is a significant peak in the crawling speed (denoted by body lengths per second, BL/s) of the soft robots owing to the PVCVAg actuator's resonance (Figure S10, Supporting Information), and the peak frequency is shifted as the voltage changes (Figure 3B; Figure S11, Supporting Information). The effect of the single‐layer thickness of the PVCVAg actuators on the crawling speed of the soft robot is shown in Figure 3C. For PVCVAg actuators with different single‐layer thicknesses, each test frequency is selected based on the resonant frequency obtained at the lowest driving voltage at which the robot can achieve motion. The soft robots with a single‐layer thickness of 30 µm start to move at 72.5 V, achieving a speed of 0.2 BL s^−1^ at 92.5 V and reaching up to 1.18 BL s^−1^ at 192.5 V. For the robots with a single‐layer thickness of 75 µm, the crawling motion begins ≈160 V, exhibiting a speed of 0.51 BL s^−1^ at 210 V and 2.94 BL s^−1^ at 460 V. The PVCVAg actuator with a single‐layer thickness of 75 µm is used in the following cases owing to its better stability. Overall, the designed rolled PVCVAg actuator enables the miniaturized soft robot to move rapidly using an ultra‐low electric field/voltage (Figure 3D; Figure S12, Supporting Information).

Next, the locomotion capabilities of the soft robots are demonstrated under different conditions. The robots demonstrate the ability to crawl on surfaces with different roughness profiles *Sa* (Figure 3E). Notably, the paper surface with a *Sa* of 2.98 µm is found to allow the highest crawling speed. Furthermore, the locomotion of soft robots on slopes (1°–11°) is also evaluated, as shown in Figure 3F. The robots can still achieve a speed of more than 1 BL s^−1^ when climbing a slope of 11° (Movie S2, Supporting Information). Moreover, the robots can carry a load of up to 6g (≈5 times the weight of a single robot) with a speed of 0.12 BL s^−1^ at 250 V (Figure S13, Supporting Information), demonstrating the potential for integrating various tools for different missions. Durability testing for robot applications is also very important. Therefore, we conducted 100 on‐off cycle tests on the robot along a designed path, as shown in Figure 3G. The robot completes an entire route in ≈20–25 s and then stops for 25 s before proceeding to the next on‐off cycle test. The test frequency was around the resonance frequency, which is 370 Hz. After 100 on‐off tests, the PVCVAg actuator underwent ≈920 000 actuation cycles. However, the speed of the robot did not deteriorate significantly, only decreasing from an average speed of 1.57 BL s^−1^ in the first ten cycles to 1.39 BL s^−1^ in the last ten cycles, with a decrease of ≈11%. Environmental durability was also tested, and the robot's speed did not significantly deteriorate after UV exposure or immersion in water (Note S3 and Figure S14, Supporting Information). In addition, the robots exhibited high physical durability, displaying stable performance after being subjected to different types of external shocks. The crawling speed remains almost unchanged (within a 3% difference) after significant bending or heavy hammering, as shown in Figure 3H and Movie S3 (Supporting Information). Power consumption is also evaluated for guidance of real‐world deployment. Under a given sinusoidal bias voltage signal (high level 250 V, low level 0 V, frequency 350 Hz), the robot consumes 168 mW of power with a movement speed of ≈1.07 BL s^−1^ after stabilization (Note S4 and Figure S15, Supporting Information).

### 2.3 Electrostatic Adhesion

**Figure 4:**
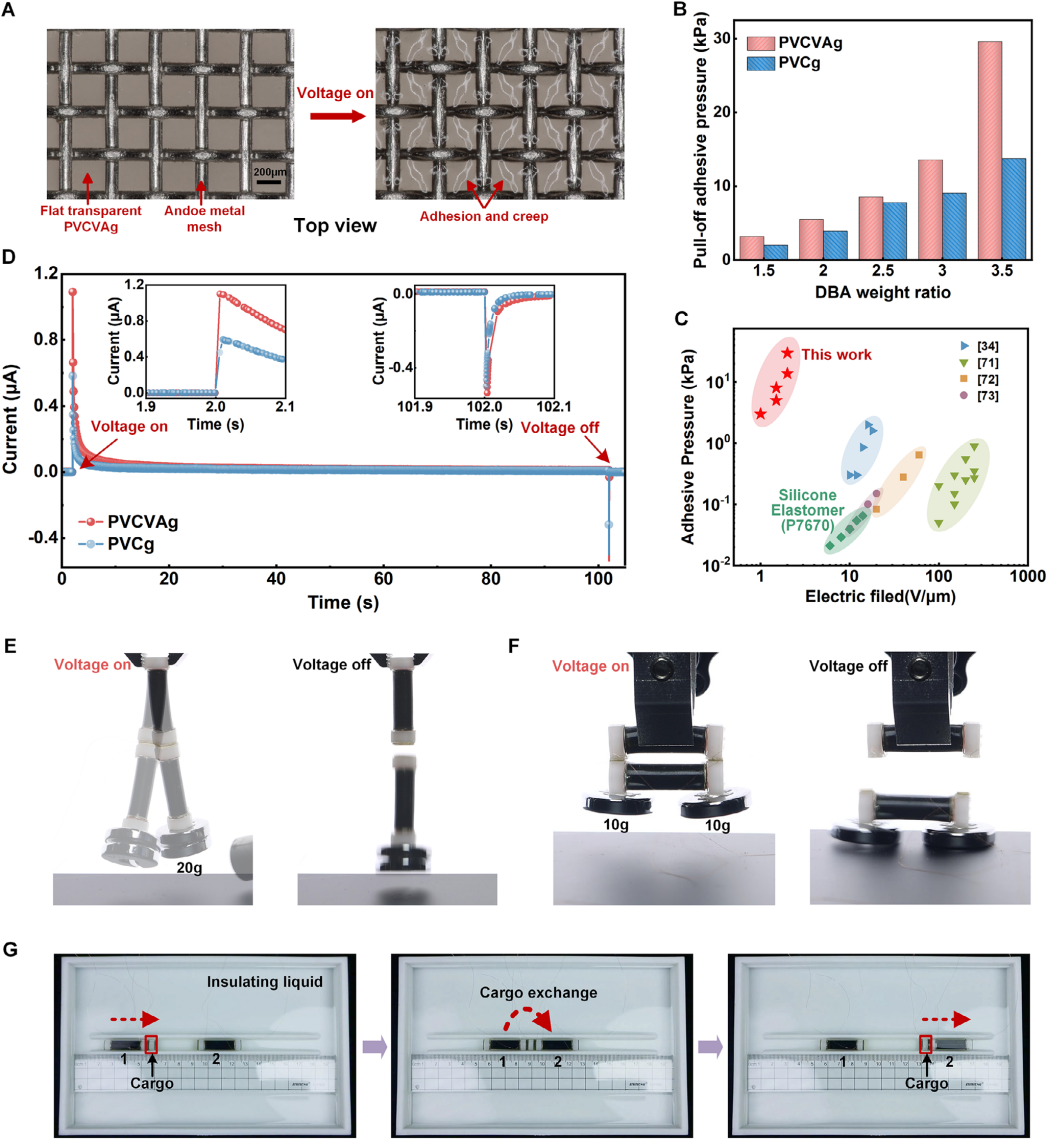
Electrostatic adhesion. A) Illustration of electrostatic adhesion between PVCVAg and the metal mesh. B) Pull‐off Adhesive pressure for PVCg and PVCVAg with different DBA weight ratios under the electric field of 2 V µm^−1^. C) The reported adhesive pressure of similar adhesion structures versus the electric field. D) Current response per square millimeter of PVCg‐based and PVCVAg‐based electrostatic adhesion structures during electro‐adhesion (DBA weight ratio is 3.5; electric field is 2 V µm^−1^). The insets show enlargements of the curve where the voltage is turned on and off. E,F) Longitudinal and parallel electrostatic adhesion demonstration for the soft robots. G) Image of the electrostatic adhesion for cargo exchange in an insulating liquid. Electrostatic adhesion structures are installed at the ends of the cargo and Robot 1, and 2.

For traditional PVCg, an electrostatic adhesive force is generated at the interface between the polymer and the anode under an electric field, which is known as an anodic adhesion phenomenon. Similarly, PVCVAg exhibits an even stronger adhesive force with the anode, making it highly suitable for miniaturized low‐voltage electrostatic adhesion structures. Studies have shown that this adhesion is related to the migration of plasticizers combined with negative charges, resulting in the formation of a negatively charged S‐R layer near the anode.^[^ ^68^, ^69^, ^70^ ^]^ Negative charges in the S‐R layer induce electrostatic adhesion with the anode, allowing the PVCVAg to deform and wrap around the anode, as shown in **Figure** **4**A and Movie S4 (Supporting Information). Here, metal meshes are chosen as the anode to prevent the formation of van der Waals forces, which may hinder subsequent disconnection. The general structure consists of two parts: PVCVAg with the cathode (flexible CNT electrodes) and the rigid metal mesh anode. Such connection structures can be easily sized to less than 5 mm.

Using an electric field of 2 V µm^−1^, the pull‐off adhesive pressures based on PVCg/PVCVAg with different DBA contents are determined (Figure 4B; Figure S16, Supporting Information). Regardless of the DBA content, the pull‐off adhesive pressure of PVCVAg is significantly larger than that of PVCg (a maximum 2.15‐fold difference). Specifically, for the DBA weight ratio of 3.5, the pull‐off adhesive pressure of PVCVAg can reach up to 29.6 kPa, and shear adhesive pressure is 14.84 kPa (Figure S17, Supporting Information), showcasing an order‐of‐magnitude enhancement in adhesive pressure while the required electric field is reduced by an order‐of‐magnitude compared to dielectric elastomer adhesives and traditional electrostatic adhesion structures (Figure 4C).^[^ ^34^, ^71^, ^72^, ^73^ ^]^ Under an electric field of 2 V µm^−1^, the current response is measured during adhesion for PVCVAg and PVCg with a DBA weight ratio of 3.5 (Figure 4D). A positive peak current appears initially, followed by a dramatic decrease and leveling off, resulting from the formation of the S‐R layer. Additionally, a temporary negative peak current occurs when the voltage is turned off, which is due to the release of charge from the anode. Both the positive and negative current peaks of PVCVAg are larger than those of PVCg, indicating a greater adhesive force for PVCVAg. The stable current response per square millimeter of adhesion area for PVCVAg before the voltage turn‐off in Figure 4D is only 12 nA, demonstrating an acceptable energy consumption for robotic applications. A simple RC circuit model was used to simulate its current response in order to evaluate the dynamic changes in the overall resistance and capacitance of the material over time (Note S5 and Figure S18, Supporting Information).

The capability of PVCVAg‐based adhesion structures is further validated by carrying different masses (Figure 4E,F). In Figure 4E, the longitudinal adhesion between the robots can withstand an external mass of 20 g (17.8 times the weight of a single robot and over 730 times the weight of electro‐adhesion structures) without detaching when subjected to dynamic shocks (Movie S5, Supporting Information). Similarly, in Figure 4F, the parallel adhesion between the robots can also support a mass of 20 g (Movie S6, Supporting Information). Additionally, we investigate the electrostatic adhesion of PVCVAg in insulating liquids, as shown in Figure 4G and Movie S7 (Supporting Information). The detailed setup is described in Note S6 (Supporting Information). The cargo is initially adhered at the front end of Robot 1, and is moved forward by Robot 1 until making contact with the back end of Robot 2. Then, the cargo is disconnected from Robot 1, and the opposite side of the cargo is adhered to the back end of Robot 2, achieving detachment and re‐adhesion in an insulating liquid.

### 2.4 Steering Motion and Self‐reconfiguration for Multiple Robots

**Figure 5:**
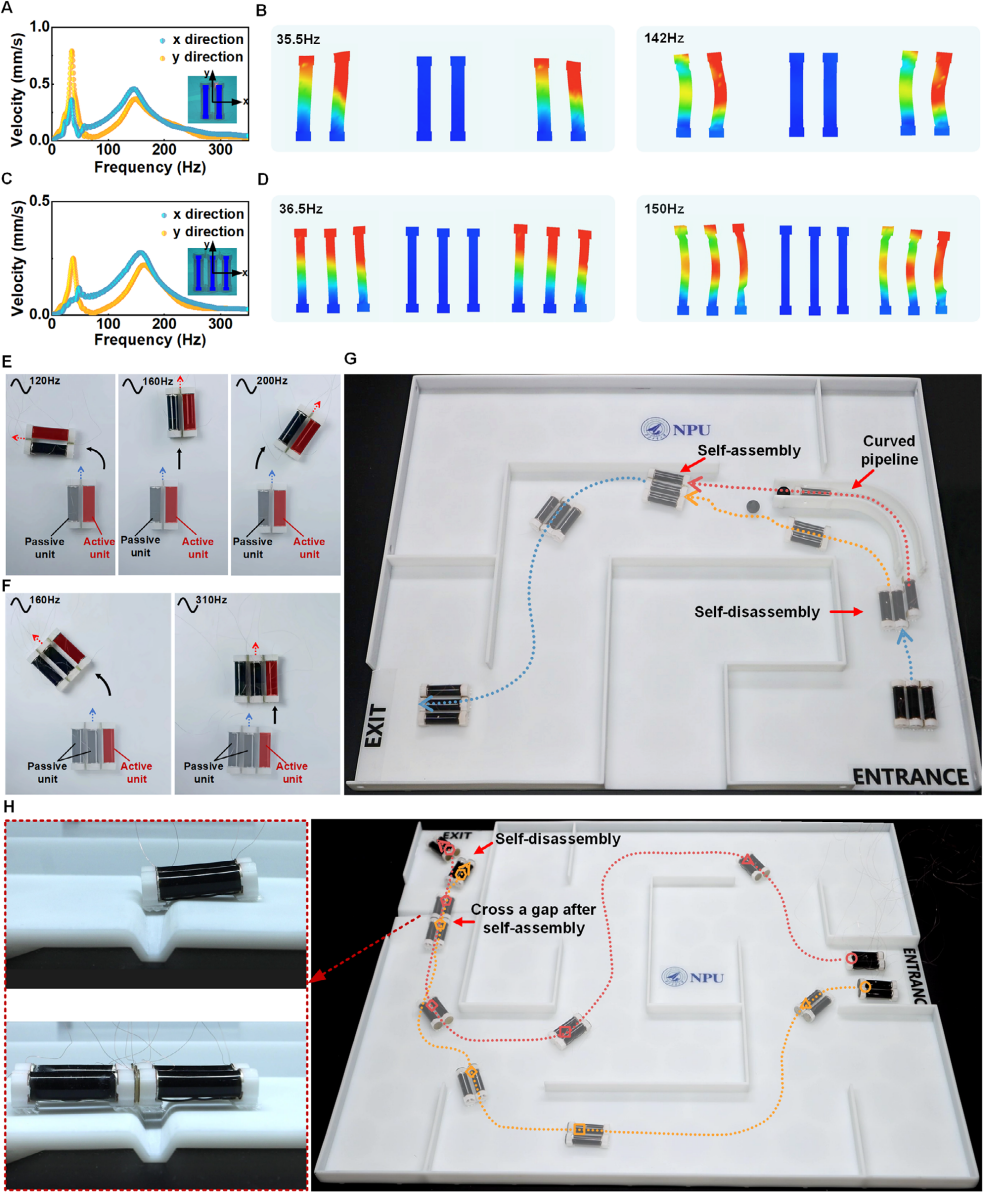
Steering motion and self‐reconfiguration for multiple robots. A,B) Velocity‐frequency response curve and the dynamic responses for two‐unit parallel robots (lower ends are fixed). C,D) Velocity‐frequency response curve and the dynamic responses for three‐unit parallel robots (lower ends are fixed). E) Different motion of two‐unit parallel robots is achieved when only the right unit is actuated under different voltage frequencies. F) Different motion of three‐unit parallel robots is achieved when the rightmost unit is actuated under different voltage frequencies. G) Image showing the use of self‐reconfiguration to operate in a narrow pipeline. H) Image showing the use of self‐reconfiguration to cross the gap.

The rapid electrostatic adhesion and release of PVCVAg are useful for the self‐reconfiguration of multiple miniaturized robots. Various configurations can be achieved for different applications by designing different PVCVAg‐based electrostatic adhesion structures. Here, parallel self‐reconfiguration and longitudinal self‐reconfiguration forms are demonstrated (Figure S19 and Note S7, Supporting Information). Parallel self‐reconfiguration allows two or more soft robots to be connected side by side, enabling them to accomplish complex tasks that are difficult for a single robot, such as turning or carrying loads. Considering the complex nonlinear dynamic responses, the crawling performance of parallel self‐reconfigurable robots does not simply depend on which robot is driven, but also on the applied driving frequency. To determine the relationship between the motion and the driving frequency of self‐reconfigurable soft robots, the dynamic responses of two‐unit parallel robots (**Figure** **5**A,B; Movie S8, Supporting Information) and three‐unit parallel robots (Figure 5C,D; Movie S9, Supporting Information) are examined, with only the rightmost unit being actuated. The velocity magnitude measured at the center of the upper end‐caps of the rightmost units for two‐ and three‐unit parallel robots is shown in Figure 5A,C. Notably, the velocity magnitude shows two major peaks at 35.5 and 142 Hz for two‐unit parallel robots and at 36.5 and 150 Hz for three‐unit parallel robots. At these peak frequencies, the self‐reconfigurable robots show different and unanticipated dynamic responses (Figure 5B,D), which are far from their static deformation. Accordingly, the steering motion modes of the self‐reconfigurable robots can be reversed at different frequencies. As shown in Figure 5E and Movie S10 (Supporting Information), when only the right unit is actuated, the two‐unit parallel robots can perform a sharp left turn at 120 Hz, go straight at 160 Hz, and turn right at 200 Hz. Similarly, the three‐unit paralleled robots exhibit analogous behavior when only the rightmost unit is actuated, turning left at 160 Hz and going straight at 310 Hz (Figure 5F; Movie S11, Supporting Information). However, due to the severe restriction of the two passive units, the right turn is not observed at any frequency. Benefiting from the frequency‐dependent characteristics of the self‐reconfigurable soft robots, damage to a single robot unit is not expected to have a catastrophic impact on the overall performance.

The swarming behavior of self‐reconfigurable robots allows for a greater range of movement. First, we demonstrate the assembly and disassembly of three‐unit parallel robots in a maze involving a curved narrow pipeline (Figure 5G). The three‐unit parallel robot starts at the entrance of the maze, and aligns the rightmost unit with the inlet of the curved pipeline (width of 1.2 cm) through appropriate motion operations. Afterward, by switching off the electric adhesion of the rightmost unit, the single robot disconnects from the other two and navigates the curved narrow pipeline (e.g., for waste cleaning). Simultaneously, the remaining two‐unit parallel robot moves toward the exit of the curved narrow pipeline, taking the outside path. Then, the single robot exits from the pipeline and reconnects with the two‐unit parallel robot. Finally, the self‐reconfigured three‐unit parallel robot moves to the exit of the maze (Movie S12, Supporting Information). In addition, the longitudinal self‐reconfiguration of multiple robots is performed to cross a gap, as shown in Figure 5H and Movie S13 (Supporting Information). Two groups of two‐unit parallel robots enter another maze and stop in front of a gap via different paths. The individual two‐unit parallel robots fail to cross the gap because of the limited robot length. However, the separate two‐unit parallel robots can connect longitudinally, forming an extended integral unit that passes over the gap successfully (Movie S14, Supporting Information). Afterward, the unified robots can be separated by turning off the adhesion voltage. These results indicate that the functionality and flexibility of miniature robots can be significantly improved by self‐reconfiguration based on electrostatic adhesion. The achievement of self‐reconfiguration in robots with a dimensional limitation below 1 cm is expected to facilitate the advancement of robotic swarms (Table S1, Supporting Information).

### 2.5 Self‐Heating and Motion in Freezing Environments

**Figure 6:**
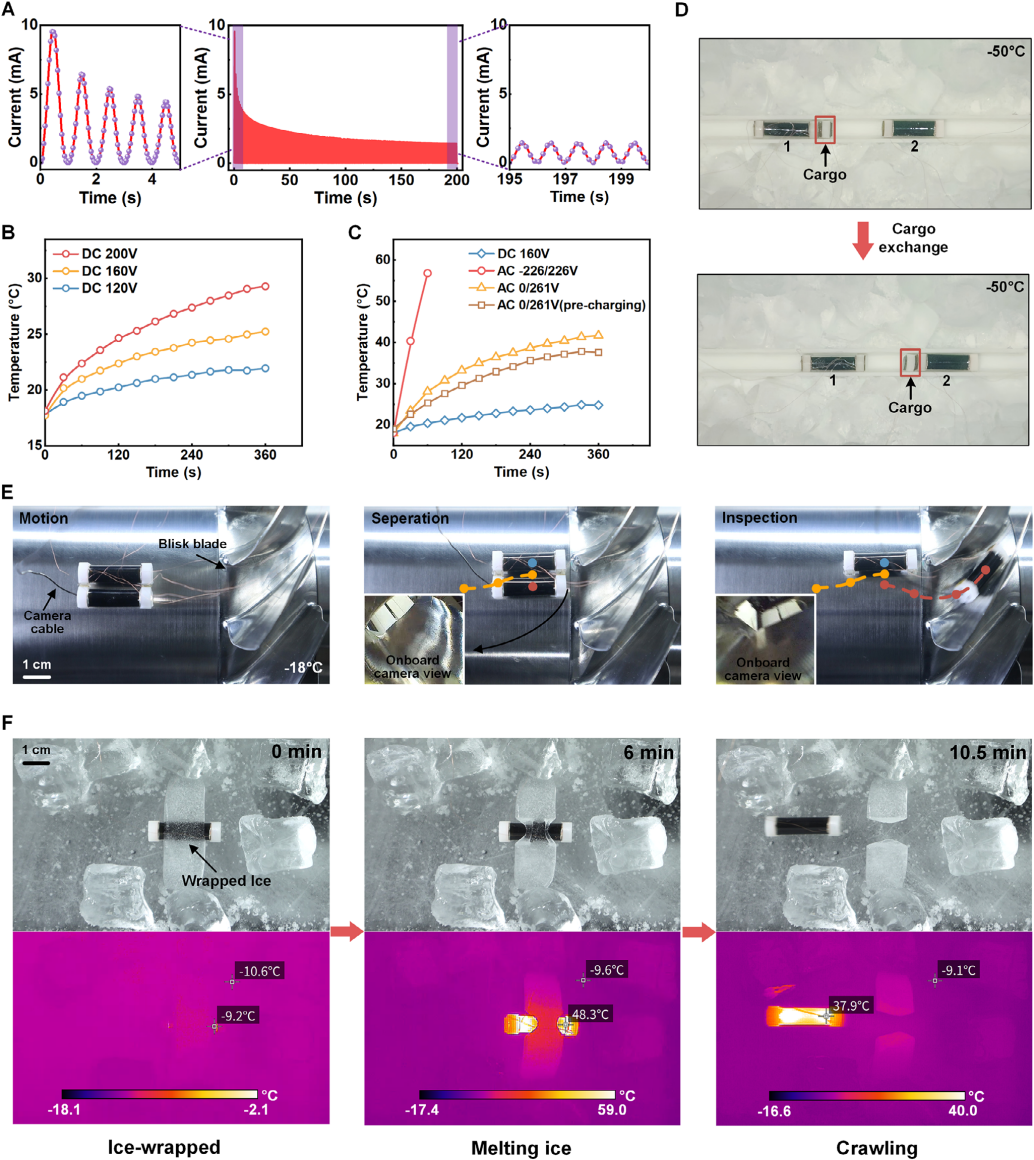
Self‐heating and operation in freezing environments. A) Dynamic evolution of the current response for the rolled PVCVAg actuator. B) Temperature change for rolled PVCVAg actuators at different DC voltages. C) Temperature change for rolled PVCVAg actuators using different voltage waveforms. D) Motion and adhesion at −50 °C. E) Motion, separation, and inspection on the aero‐engine Stage 1 blisk blade at −18 °C. F) Melting wrapped ice by controlled self‐heating and moving forward in a freezing environment.

The dynamic evolution of S‐R layer and current response under the electric field for PVCVAg provides guidance for the suppression and utilization of self‐heating. Here, the current response of the rolled PVCVAg actuator from 0 to 200 s is investigated (**Figure** **6**A), using a sinusoidal bias voltage (high level 150 V, low level 0 V, 1 Hz). The change in current is pronounced, with a continuous decrease from ≈10 mA to 1.7 mA. The current in the adhesion process mentioned in Figure 4D has the same tendency to decrease and then stabilize, even though it is under a DC voltage. This indicates that the migration of the plasticizers combined with negative charges in PVCVAg under an electric field is not instantaneous, and the formation of the S‐R layer takes at least a few seconds (Figure S20‐I, II, Supporting Information). Similarly, the disappearance of the S‐R layer is not immediately completed after the removal of the voltage (Figure S20‐III, Supporting Information), and thus, the stability of PVCVAg is gradually achieved after applying repeated sinusoidal voltage.

Heating regulation is a highly desirable technology for soft robots, enabling effective and wide‐scale environmental interaction. For the heating regulation of PVCVAg, different voltage loading strategies are implemented, either suppressing or amplifying the heat generation. First, the effect of a DC voltage on the heating performance of the rolled PVCVAg actuator is investigated (Figure 6C), showing that a higher voltage induces a higher temperature. The voltage waveform also largely determines the level of heat generation, and we compared several waveforms with the same value of the root mean square, including a DC voltage (160 V, named DC 160 V), sinusoidal voltage (amplitude 226 V, frequency 350 Hz, named AC −226/226 V) and sinusoidal bias voltage (high level 261 V, low level 0 V, frequency 350 Hz, named AC 0/261 V), as shown in Figure 6D and Figure  S16. Under DC 160 V, the rolled PVCVAg actuator exhibits minimal heat generation owing to the rapid balance of the S‐R layer, facilitated by the unidirectional movement of polarized plasticizers combined with negative charges in an unchanged electric field. In contrast, a sinusoidal voltage (AC −226/226 V) greatly increases the heat generation. The periodically inverted electric field imposed by the sinusoidal voltage makes the migration of plasticizers/charges more intense and the formation of a stable S‐R layer more difficult. For the sinusoidal bias voltage (AC 0/261 V), the cyclic variation of the electric field value between zero and the maximum value causes the S‐R layer to form and dissipate within each voltage cycle. The S‐R layer spontaneously dissipates owing to differences in the plasticizer concentration gradients, and the dissipation rate likely differs from the formation rate. As a result, the S‐R layer gradually establishes an equilibrium under the sinusoidal bias voltage. In addition, we found that pre‐charging can further reduce the heat generation of the rolled PVCVAg actuator when the same sinusoidal bias voltage is applied. Pre‐charging refers to the prior application of a lower voltage to partially form the S‐R layer beforehand (Figure S21, Supporting Information). For example, before applying a sinusoidal bias voltage with a high level of 261 V (AC 0/261 V), the high level is initially set to be 37.3 V. Then, every 10 s, the high level increases by 37.3 V until reaching 261 V, during which the frequency and the low level remain constant. The 60 s precharging process reduces the final temperature of the actuator by ≈20%, as shown in Figure 6D.

On this basis, the actuation and adhesion capabilities of robots in extremely cold environments (−50 °C) are demonstrated (Figure 6E). The results show that, the normal actuation and adhesion can be ensured in the extremely low‐temperature environment, realizing the transfer and exchange of target cargo (Movie S15, Supporting Information). Robotic interventional operations on spatially‐constrained equipment in freezing environments impose stringent demands on robotic systems. Here, Figure 6F and Movie S16 (Supporting Information) demonstrate the coordinated motion, module separation, and individual inspection of the soft robots on the aero‐engine Stage 1 blisk blade at −18 °C, revealing the application potential of such robots in complex and confined spaces in freezing environments. In order to clearly observe the internal morphology and damage of the blisk blade, a miniature camera is equipped within the front‐end cap of the soft robot. Furthermore, the self‐heating function is verified using a robot wrapped in ice in a freezing environment (Figure 6G; Movie S17, Supporting Information). Unbiased sinusoidal voltage is first applied to quickly generate a lot of heat and bring the robot above the freezing point. After the ice on the robot has melted, the sinusoidal bias voltage is adjusted to regulate heat generation and actuate the robot moving on the ice surface.

## 3 Conclusion

In this work, we have demonstrated the feasibility of multi‐functional PVCVAg and their application in soft robotics. Specifically, by introducing PVCVA into PVCg, the electromechanical properties are enhanced while significantly mitigating the heat generation and premature breakdown. Our modification strategy and emphasis on stability provide new insights for future material design and application potential of PVCg. We have also explored the rapid fabrication process for rolled PVCVAg actuators and applied them to robotic actuation. The robots can crawl on surfaces with different roughnesses, slopes, and various media, and withstand external shocks. The electrostatic adhesion structure based on PVCVAg and metal mesh is also investigated. PVCVAg has a higher adhesive pressure than traditional PVCg and other electrostatic adhesion structures, enabling the successful application of electrostatic force in the connection of self‐reconfigurable robots for the first time. Benefiting from the miniaturization of the PVCVAg‐based adhesion structure, the robot is much smaller and lighter than most existing macroscopic self‐reconfigurable robots. Furthermore, the frequency‐dependent dynamic characteristics of the self‐reconfigurable soft robot are also investigated for enhanced movement and control. As a proof‐of‐concept application, the self‐reconfiguration of swarmed soft robots by electrostatic adhesion is demonstrated and leveraged to inspect narrow pipelines and cross gaps. In addition, the electromechanical evolution of PVCVAg is studied, and different voltage stimulation strategies are applied for suppressing or amplifying heat generation. Although we significantly reduce the heat generation by introducing PVCVA, pre‐charging is found to further decrease the actuator's heat generation. And a sinusoidal voltage signal with a repeatedly reversing electric field can generate a significant amount of heat at a low voltage. The adjustable self‐heating properties enable our robots to operate in freezing environments and even melt ice. The developed PVCVAg material with advanced electroactive properties and the design of soft robots with multiple task execution capabilities provide new insights into broadening the diversified actuation and application of soft robots in extreme and complex conditions.

## Conflict of Interest

The authors declare no conflict of interest.

## Author Contributions

C.W. and J.Z. (Junshi Zhang) contributed equally to this work. C.W., J.Z. (Junshi Zhang), and L.L. conceived the concept and designed the experiments. C.W., H.Y., K.W., and Y.H. carried out experiments and collected the data. C.W., J.Z. (Junshi Zhang), and L.L. completed the figure depicture. C.W. drafted the manuscript. J.Z. (Junshi Zhang), L.L., M.C., Z.Z., J.Z. (Jihong Zhu), W.Z., Z.W., and J.L. revised the manuscript. J.Z. (Jihong Zhu), W.Z., Z.W., and J.L. supervised the project. All authors discussed the results and commented on the manuscript.

## Supporting information

Supporting Information

Supplemental Movie 1

Supplemental Movie 2

Supplemental Movie 3

Supplemental Movie 4

Supplemental Movie 5

Supplemental Movie 6

Supplemental Movie 7

Supplemental Movie 8

Supplemental Movie 9

Supplemental Movie 10

Supplemental Movie 11

Supplemental Movie 12

Supplemental Movie 13

Supplemental Movie 14

Supplemental Movie 15

Supplemental Movie 16

Supplemental Movie 17

## Data Availability

All data are available in the paper and supplemental information and/or from the corresponding authors upon reasonable request.
