# Supplementary material for: Vinyl Acetate‐Enhanced Polyvinyl Chloride Gel with High Electroadhesion and Self‐Heating‐Tunability for Soft Robots in Freezing Environments
Source: Adv Sci (Weinh). 2025 Aug 11;12(41):e07757. doi: 10.1002/advs.202507757 (PMC12591209; doi:10.1002/advs.202507757)
Supplement: Supplementary file 1 — Supporting Information [file ADVS-12-e07757-s006.pdf]

Supporting Information

Vinyl Acetate-Enhanced Polyvinyl Chloride Gel with High Electroadhesion and Self-Heating-Tunability for Soft Robots in Freezing Environments

Chang Wei, Junshi Zhang, Lei Liu*, Han Yan, Kaijun Wang, Yuzheng He, Minchao Cui, Zicai Zhu, Jihong Zhu*, Weihong Zhang, Zuankai Wang, Jian Lu**

C. Wei, J. Zhang, L. Liu, H. Yan, K. Wang, Y. He, M. Cui, J. Zhu, W. Zhang
School of Mechanical Engineering
Northwestern Polytechnical University,
Xi'an, China
E-mail: junshi.zhang@nwpu.edu.cn, J. Zhang; liulei2022@nwpu.edu.cn, L. Liu;
jh.zhu@nwpu.edu.cn, J. Zhu.

C. Wei, Z. Wang
Department of Mechanical Engineering
The Hong Kong Polytechnic University
Hong Kong SAR, China

Z. Zhu
School of Mechanical Engineering
Xi'an Jiaotong University
Xi'an, China

J. Lu
Department of Mechanical Engineering
City University of Hong Kong
Hong Kong SAR, China
E-mail: jianlu@cityu.edu.hk, J. Lu

Supplemental Notes

Note S1. Experimental Section

Materials: PVC powder (Mw 275,000, CAS: 9002-86-2) and PVCVA powder (Mw 115,000, CAS: 9003-22-9) are purchased from Scientific Polymer Products. Tetrahydrofuran (THF) and dibutyl adipate (DBA) are purchased from Macklin. The ultrapure single-walled CNT aqueous dispersion (0.2 wt. %) is purchased from Timesnano. Silver epoxy adhesive is MG Chemicals 8331D. All the commercially available reagents are used without further purification.

Fabrication of PVCg, PVCVAg and CNT Electrodes: Different gels were produced based on the various weight ratios of PVC powder, PVCVA powder, and DBA. The PVC/ PVCVA powder were dissolved in THF. After obtaining a uniform solution, DBA was added and the mixture solution was stirred at 1000 rpm for 6 h at 50 °C. Then, doctor blade coating method was used to fabricate PVCg or PVCVAg with a thickness below 400 μm. The solution was apply onto the Polyethylene Terephthalate (PET) film (thickness 300 μm) at a temperature of 42 °C. The final thickness of PVCg/PVCVAg can be adjusted by the gap between the film applicator and the PET. After standing for 0.5-1 h (depending on the thickness of gel), there was no THF residue remaining in the gel. Besides, casting method was used to fabricate the gel with a thickness over 400 μm, during which the mixture solution was poured into petri dishes and left for 5 days for THF evaporation.

The CNT original solution was initially diluted 1000 times, and then the diluted solution was taken with different volumes based on the required surface density of the CNT electrode. A layer of CNT film on the filter membrane (Mixed cellulose esters, 0.1 μm, Shanghai XingYa) was produced by vacuum filter. CNT electrodes with different shapes could be fabricated by pad printing the CNT film onto the PVCg/PVCVAg surface with various masks (PET film, 50 μm). Four-point probes (RTS-8, GuangZhou 4 Probes) were used to measure the surface resistance of CNT electrodes.

Characterizations of PVCg/PVCVAg: For the measurement of dielectric constant, the samples were cut into a circular shape with a diameter of 38 mm and thickness 250 μm. The tests were conducted based on the LCR Meter (IM3536, HIOK) with a dielectric test fixture (16451B, Agilent). We performed relative dielectric constant measurements across a frequency range of 20-10^6 Hz. The storage modulus G′ and loss modulus G″ were measured by Rotational rheometer (Kinexus Lab+, Netzsch). The test smaples of PVCg/PVCVAg are 20 mm in diameter and 2mm thick.

Preparation of the Rolled Actuators and Electroactive Response Measurement: For planar actuators, the film with an initial thickness of 250 μm is pre-stretched by 20% and fixed between two annular rigid PMMA frames with an inner diameter of 60 mm. CNT electrodes with a 40-mm diameter was coated on both sides of the film. The breakdown strength of PVCg/PVCVAg was tested using a high-voltage amplifier (Trek 10/10B) based on the planar actuator. A DC voltage of a ramping rate of 200 V/s was applied until the voltage drops sharply.

The steps in manufacturing the cylindrical PVCg/PVCVAg actuators are as follows: (i) A certain amount of PVC/PVCVA powder were dissolved in THF. After obtaining a uniform solution, DBA was added and the mixture solution was stirred at 1000 rpm for 6 h at 50 °C to obtain the precursor. The precursor can remain stable for an extended period by sealing the beaker and keeping stirring at ambient temperature. (ii) Use an automated film applicator (HF-Kejing, MSK-AFA-H600A) to apply the precursor onto a 300μm-thick Polyethylene Terephthalate (PET) film substrate (Figure. S6A). Set the applicator temperature to 42°C and blow hot air with an approximate temperature of 45°C onto the latest applied layer surface (Figure. S6B) for a period of 30 minutes to 1 hour (time increasing with the thickness). Then the PVCg/PVCVAg membrane is obtained after the solvent THF has completely evaporated. (iii) The original Carbon Nanotube (CNT) solution is diluted 1000 times, and then a CNT film on the filter membrane is obtained by vacuum filtration of calculated amount of dilute CNT solution. (iv) A laser-cutting mask (PET film with a thickness of 50 μm) is placed over the PVCg/PVCVAg membrane. Using a stamping method, CNTs

from the filter membrane are transferred onto the layer, creating the desired shape with the mask (Figure. S6C). (v) Apply a new PVCg/PVCVAg membrane over the original one and CNT using a film applicator (Figure. S6D), then heat and hot air are applied to completely evaporate the THF (Figure. S6E). (vi) Subsequently, repeat the transfer of CNTs and PVCg/PVCVAg membranes until the desired number of layers is achieved. Ensure that the electrode lead-out areas are aligned in alternating layers to create an interdigitated structure (Figure. S6F). The thickness of the first and last layers is half of that of others, in order to form a layer with the same thickness. (vii) Trim the stacked layer along the perimeter of the CNT electrode into a strip, and expose the electrode lead-out areas at both ends (Figure. S6G). (viii) The strip is rolled to form a cylindrical shape, with the lead-out areas of the interdigitated electrodes exposed at both ends of the actuator (Figure. S6H). Apply silver epoxy conductive adhesive (8331D, MG Chemicals) to the ends with exposed CNTs, then cover with circular copper-clad laminate at the ends, and lead out the wires for electrical connections to finalize the fabrication of the cylindrical PVCg/PVCVAg actuators.

The static performances of rolled PVCg/PVCVAg actuators were measured by a laser sensor (LK-G80, Keyence) and a force sensor (LH-S09A3-2N, Shanghai Liheng). The actuator stood vertically and the laser sensor was placed above the rolled actuator to measure its free displacement. The force sensor was slightly in contact with the upper end of the rolled actuator to measure the blocking force. Ramp voltage is used to achieve the target voltage within 6 seconds. Dynamic displacements in actuator's resonance test were measured by highly dynamic laser sensor (optpNCDT 2300, Micro-Epsilon). The frequency started at 1 Hz and was increased by 10 Hz every three seconds. Infrared camera (SC7900, FLIR) was used to monitor the temperature, and maximum temperatures were recorded. The actuators were powered by high-voltage source, which was generated by a signal generator (DG4000) and a high-voltage amplifier (ATA-7000). Digit multimeter (DMM6500, Keithley) was used to monitor the current response. Simulink is used to simulate the current response of equivalent circuit.

Pull-off adhesive pressure tests (Figure S17B, Supporting Information) were performed on an electronic universal testing machine (CMT6000, Senstest). The PVCg/PVCVAg with CNT electrodes on one side was secured to the fixture of the testing machine with tape. The other end of the testing machine was eqipped with a metal mesh anode attached to the fixture with tape. The wires are connected to the positive and negative electrodes to make an electrical connection. Metal mesh and PVCg/PVCVAg remain parallel and intercontact with each other. After the voltage was applied, the testing machine with a moving rate of 6 N/min to stretch the adhesion structure until the failure of adhesion. The difference between the highest and lowest values of forces from the testing machine during the stretching process was the adhesive force. The adhesive pressure is obtained by dividing the adhesive force by the adhesive area (25mm×25mm, 625mm^2 in this test). Shear adhesive pressure tests (Figure S17A, Supporting Information) were performed on an home-made desktop test system (a force sensor (HANDPI HP-10) and a DC motor (NiMotion)). After the voltage was applied, the testing machine with a moving rate of 6 cm/min to stretch the adhesion structure until the failure of adhesion. The adhesive pressure is obtained by dividing the adhesive force by the adhesive area (20mm×6mm, 120mm^2 in this test). The thickness of samples used for adhesion is approximately 200 μm.

Fabrication and testing of soft robots: First, the ends of the rolled PVCVAg were glued to two resin end caps, respectively. Then, friction anisotropic feet were attached to the bottom of two end caps. Next, the metal mesh and rectangular PVCVAg (5 mm×4 mm) containing CNT electrodes on one surface were attached to the lateral sides of the end caps, respectively. The metal mesh and CNT electrodes were connected to the anode and cathode via enameled wires. The transfer area of the CNT electrode was a little smaller than the PVCVAg to avoid short circuits. The front and back sides of a robot were also attached with metal mesh and circular PVCVAg (Φ7.5mm), respectively. The thickness of PVCVAg used for adhesion is 200 μm. In this way, the miniature soft robots can achieve the assembly and disassembly with other

identical modular robots through electrostatic adhesions in the parallel and longitudinal directions. The detailed weight information is listed in Table S2 (Supporting Information).

The durability test of the robot is based on the path shown in Figure 3G. The total length of the path is 984 mm, and the crawling speed of the robot is obtained by dividing the length of the path by the time to completes the entire route.

The laser scanning vibrometer (LV-SC400-3D, Sunny Optical) was used to measure the frequency response and vibration of two-unit and three-unit parallel robots in the free-standing state. Before measurement, reflective powder was sprinkled on PVCVAg actuators of robots and reflective strips were attached to the rest of robots. The Fourier transform is applied to the obtained results from laser scanning vibrometer. The dynamic response plot at the corresponding frequency can be directly exported from the its software. The high-speed camera (Photron FASTCAM SA-X2) was used to capture images of the dynamic response.

Electronic insulating liquid (Fluorinert FC-770, 3M) was used to provide the operation environment in liquid. Lead sand is encapsulated in end caps to prevent the robot from floating in liquid test. Self-reconfigurable robots realize flexible motion in a maze based on synergistic actuation between multiple units. Motion and adhesion of robots at −50 °C were conducted in a constant temperature and humidity chamber (Yoma Scientific). Motion, separation, and inspection on the blisk blade were carried out in a low-temperature environmental room maintained at −18°C. And the blisk blade was extracted from the Stage 1 integrally blisk of the TC17 titanium alloy high-pressure compressor in an aero-engine. The display of ice-melting was performed in a −10°C refrigerated room.

Statistical Analysis: Some experimental data are presented as mean values with error bars representing the standard deviation. For these data, each measurement is conducted with $n = 3$ independent samples, and the reported values reflect the average across these replicates. Given

the limited sample size, significant differences assessment is not performed, as the results may not be statistically robust. Data processing was performed with software Origin.

Note S2. The optimal CNT electrode surface density for PVCVAg actuators

The CNT electrode surface density largely affect the electromechanical performance of cylindrical PVCVAg actuators. A low CNT surface density deteriorates the electrical conductivity, while a high one increases the stiffness because of the stiff network of CNT film. There is an optimal value for the CNT surface density, which can balance the electrical conductivity and mechanical properties. In fact, the optimal value varies with electrical field strength, as shown in Figure. S7. The maximum displacement of PVCVAg actuator at an electrical field of 5 V/μm corresponds to a CNT surface density of about 0.0185 g/mm^2. However, when the electrical field is above 6 V/μm, the optimal value of CNT surface density is offset to about 0.014g/mm^2. It is worth noting that at relatively low electric field, e.g., 3 V/μm and 4 V/μm, the displacements remain essentially unchanged when the CNT surface density is above a certain value (larger than 0.0185 g/mm^2), because the overmuch CNT network has less mechanical influence on the the bulk material at small deformations. Considering that the cylindrical actuators mostly operate between 2.5 V/μm and 7 V/μm, the CNT surface density is chosen to be 0.014 g/mm^2 subsequently.

Note S3. Environmental durability

In order to assess environmental durability, we added UV exposure testing and moisture uptake testing. For the UV test, we exposed the robot under a UV lamp (ZiGu Lighting Company) with a UV wavelength of 365 nanometers and a radiation intensity of 400.0 μW/cm^2 (approximately ten times the UV radiation intensity at noon in summer on Earth). As shown in Figure S14A, after 24 hours of exposure, the speed of the robot only decreased from 1.74 BL/s to 1.50 BL/s, a reduction of approximately 13.8%. Notably, the 3D-printed resin components at both ends of the robot exhibited noticeable color aging after the same UV exposure, but the performance degradation of the actuator was not significant. This may be because the actuator is multi-layered, with the outer

layer providing protection for the inner layers, preventing overall performance deterioration. In summary, the actuator's performance does not degrade rapidly after exposure to high-intensity UV light.

For the moisture uptake test, we placed the robot directly into a petri dish filled with water. Before each test, we dried the water on its surface in a ventilated environment. As shown in Figure S14B, even after being immersed in water for 9 hours, the robot's speed only decreased from 1.56 BL/s to 1.41 BL/s, a decrease of approximately 9.6%, which demonstrates the robot's durability in wet conditions.

Note S4. Energy consumption evaluation

Energy consumption is indeed an important indicator for robots, providing important guidance for their energy sources and circuit design. Here, we measured the current and voltage data under realistic operating conditions of the robot. The driving voltage and frequency are 250V and 350Hz, respectively, and the robot's running speed is approximately 1.07BL/s. The average electrical power consumption is calculated by integrating voltage (*V*) and current (*I*):

$$\overline{P}_{\text{in}} = \frac{1}{T_1 - T_0} \int_{T_0}^{T_1} V(t) I(t) dt \tag{S1}$$

The current data is shown in Figure S15A. Due to the progressive formation of the S-R layer, the maximum current value is high at the initial stage, then gradually decays over the next few seconds to reach a stable value. The minimum current value also decreases from close to zero to a stable negative value. The changing current peaks mean that power consumption also changes, as can be clearly seen in Figure S15B, where the average power is the power consumption average over three cycles at the corresponding time point. Power consumption gradually decreased from an initial 1077 mW to 168 mW, which is on the same order of magnitude as the power consumption of other reported dielectric elastomer actuators. The inset figures in Figure Figure S15B show that the instantaneous power at different time points also varies.

In this test, our power amplifiers (ATA-7000) have a high maximum power limit, allowing them to accurately show changes in current and power under a given voltage. However, this does not mean that such a high initial current (e.g. 17mA in Figure S15A) or power output (e.g. 1077 mW in Figure S15B) is necessary to drive the robot. Theoretically, as long as the output voltage, current, and power of the circuit can meet the requirements under stable actuation conditions (that is, after the S-R layer has been stably formed at the material level), the robot can operate. The circuit requirements for PVCVAg in a stable state are similar to those for current dielectric elastomer actuators and are expected to be achievable. In fact, in our tests, the robot can also be driven by Trek 2220 power amplifiers which can only achieve a maximum current of 10 mA. Limited current and power output only slow down the formation speed of the S-R layer and increase the time required for the actuators to reach stability, but seem to have no significant effect on its ultimate actuation performance.

Note S5. RC circuit model for PVCg/PVCVAg

An equivalent circuit model (Figure S18A) incorporating time-varying resistance and capacitance is proposed to model the current response of PVCg and PVCVAg during actuation. The time-varying resistance and capacitance are the indicators for the migration of polarized plasticizers combined with negative charges under voltage loading.

The time-varying resistance and capacitance of PVCg in Figure 4D are represented as:

$$\mathrm{R}_{\mathrm{PVCg}} = 7.1\times10^{7} - 7.1\times10^{7} / \exp((t+0.105)/9)\Omega \tag{S2}$$

$$\mathrm{C}_{\mathrm{PVCg}} = 2.0\times10^{-9} - 2.0\times10^{-9} / \exp((t+0.19)/18.5)F \tag{S3}$$

where t is the time. The current data from simulation and experiment at the moments of voltage on (1.5s - 4.5s) and voltage off (100.5s - 102.5s) are shown in Figure S18B. The simulation curve fits the experimental data trend well, proving the validity of our model. With a pulse voltage signal (400V), the current response initially exhibits a peak, which then gradually decreases to a stable level (e.g., the current between 100.5 s and 102 s) due to changes

in resistance. Then, at the instant the voltage is turned off, a transient discharge current occurs due to the properties of the capacitor.

In addition, the parameters of resistance and capacitance of PVCVAg in Figure 4D are represented as:

$$R_{PVCVAg} = 5\times10^{7} - 5\times10^{7} / \exp((t+0.14)/15)\Omega \tag{S4}$$

$$C_{PVCVAg} = 2.4\times10^{-9} - 2.4\times10^{-9} / \exp((t+0.19)/18.5)F \tag{S5}$$

The current data from simulation and experiment at the moments of voltage on (1.5s - 4.5s) and voltage off (100.5s - 102.5s) for PVCVAg are shown in Figure S18C. PVCVAg exhibits a similar current trend to PVCg because the modification of our material does not alter its basic electrical response mechanism, which is the source of the difference of PVCg-based material and other EAP materials. However, both the positive and negative current peaks of PVCVAg are larger than those of PVCg, indicating a greater adhesive force for PVCVAg.

The time-varying resistance and capacitance of PVCVAg in Figure 6A are represented as:

$$R = 1\times10^{5} - 1\times10^{5} / \exp((t+4.9)/30)\Omega \tag{S6}$$

$$C = 4.87\times10^{-11} - 4.87\times10^{-11} / \exp((t+6)/30)F \tag{S7}$$

Based on the established equivalent circuit, the current response of PVCVAg can be simulated (Figure S18D), exhibiting good agreement with the experimental measurements.

In summary, the RC model provides a direct understanding of the electrical behavior of PVCg-based materials under an electric field, which can guide our design and application (e.g., heat control and potential circuit design). However, this model is somewhat superficial and lacks precision. Further research may focus on accurately fitting the electrical parameter variations over time for the S-R layer and bulk layer separately, as well as investigating the impact of different waveforms.

Note S6. Design of electrostatic adhesion structures working in insulating liquid

For the demonstration of connection and disconnection properties by electrostatic adhesion in insulating liquid (Figure. 4G), the PVCVAg films with the CNT cathodes are attached at the front end of the Robot 1 and the back end of the Robot 2. PVCVAg films are in a circular shape with a diameter of 7.5 mm and a thickness of 200 μm. The CNT electrodes are transferred on one side of the PVCVAg film, with a diameter of 6 mm. Two circular metal meshs with diameters of 7.5 mm are attached at the both sides of the cargo. Thus, both the front end of Robot 1 and the back end of Robot 2 have paired electrostatic adhesion structures with the cargo.

Note S7. Design of electrostatic adhesion structures for self-reconfiguration of mutiple insect-scale soft robots

For the parallel and longitudinal self-reconfiguration forms demonstrated in Figure 5, electrostatic adhesion structures are installed at the side and front/back surfaces of two end caps of the insect-scale robots. Specifically, for the parallel self-reconfiguration, the side surfaces of two end caps are attached with PVCVAg film/CNT cathode or metal mesh anode (Figure. S18(i)), respectively. The PVCVAg film and metal mesh anode have an area of 5 mm×4 mm and the CNT cathode has an area of 4 mm×3 mm. Once the PVCVAg film/CNT cathode on the side surface of one robot is aligned in contacted with the metal mesh anode on the side surface of the other robot, electrostatic adhesion can be actived and the parallel self-reconfiguration can be achieved. Similarly, for the longitudinal self-reconfiguration, the front and back sides of a robot are attached with PVCVAg film/CNT cathode and metal mesh anode (Figure. S18 (iii)), respectively. The areas of PVCVAg film and metal mesh anode are in a circular shape with a diameter of 7.5 mm. The diameter of CNT electrodes is 6 mm. If the front end of one robot (with PVCVAg film/CNT cathode) and the back end of the other robot (with metal mesh anode) is contacted, the electrostatic adhesion and corresponding longitudinal self-reconfiguration can be achieved.

Supplemental Figures

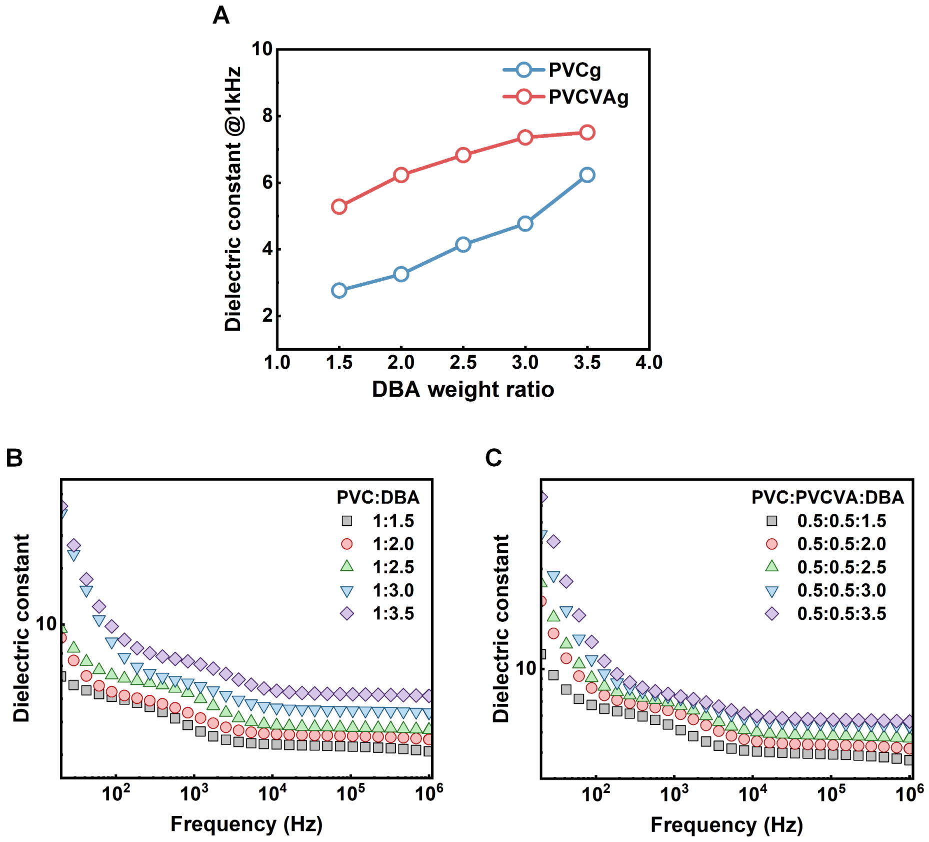

Figure S1. (A) Dielectric constant of PVCg and PVCVAg with different weight ratios of DBA. For PVCg, the DBA weight ratio is the weight of DBA relative to PVC. For PVCVAg, the DBA weight ratio is the weight of DBA relative to PVC and PVCVA together, and the weight ratio of PVC and PVCVA is 0.5:0.5. (B and C) Dielectric constants of PVCg with different weight ratios of DBA and PVCVAg with different weight ratios of DBA under frequencies of 20-10^6Hz.

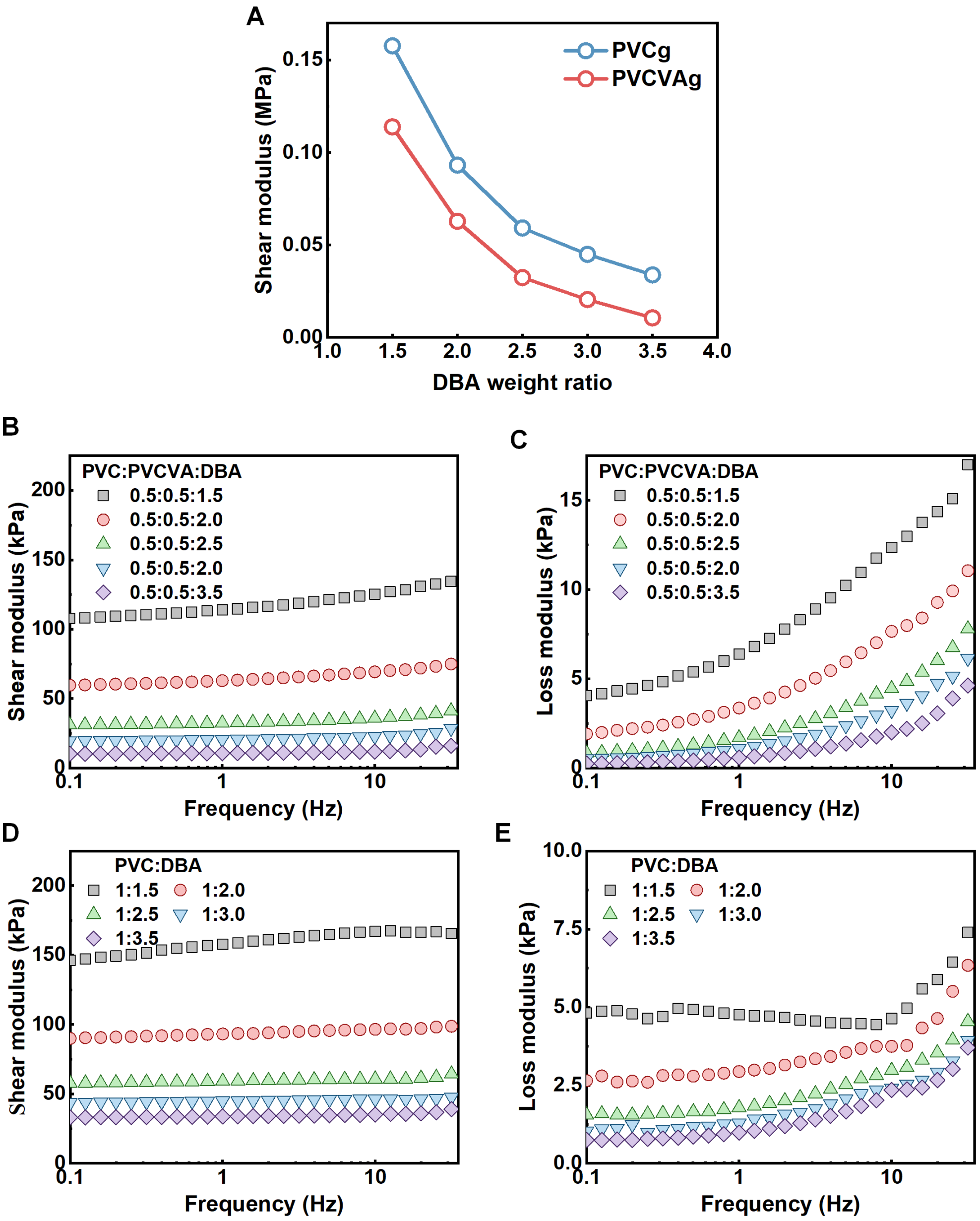

Figure S2. Mechanical properties of PVCg and PVCVAg. (A) Shear modulus of PVCg and PVCVAg with different weight ratios of DBA. (B and C) Shear modulus and loss modulus of PVCVAg with different weight ratios of DBA under frequencies of 0.1-30 Hz. (D and E) Shear modulus and loss modulus of PVCg with different weight ratios of DBA under frequencies of 0.1-30 Hz.

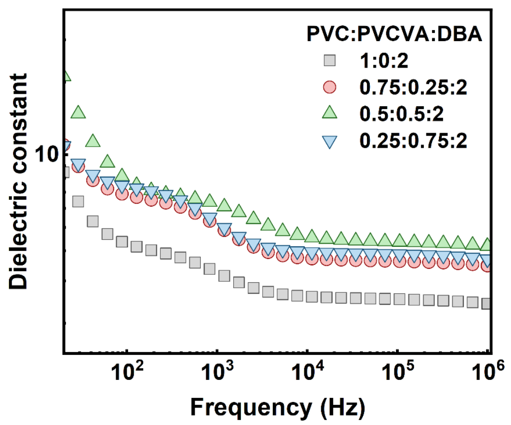

Figure S3. Dielectric constants of PVCVAg with different weight ratios of PVC and PVCVA under frequencies of 20-10^6Hz.

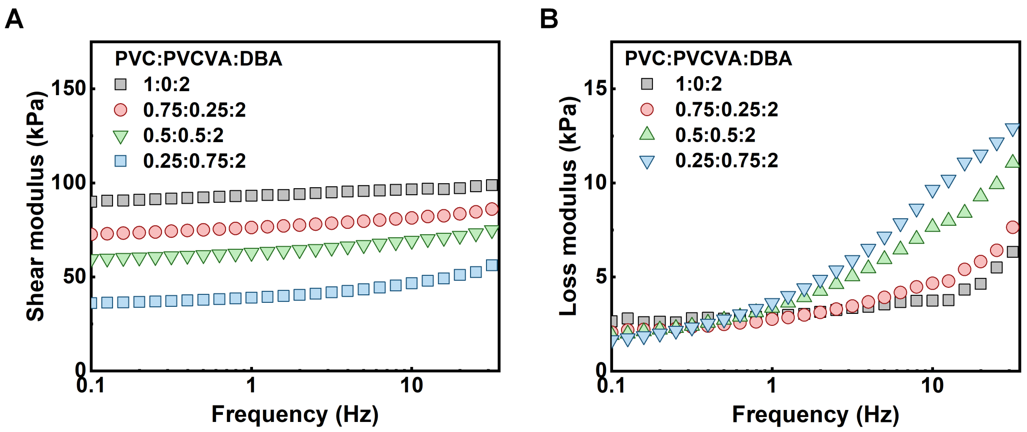

Figure S4. Shear modulus and loss modulus of PVCVAg with different weight ratios of PVC and PVCVA under frequencies of 0.1-30 Hz.

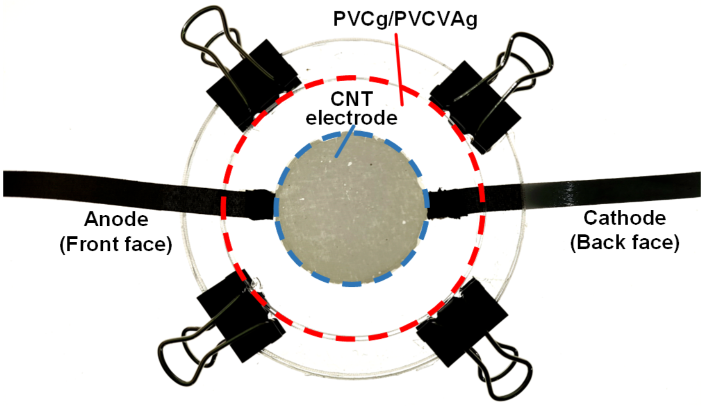

Figure S5. Illustration of planar PVCg/PVCVAg used for heat generation test. CNT electrodes are transferred on both sides of the PVCg/PVCVAg film, respectively. A pre-stretch of 20% is applied to prevent initial deformation induced by self-gravity of the film.

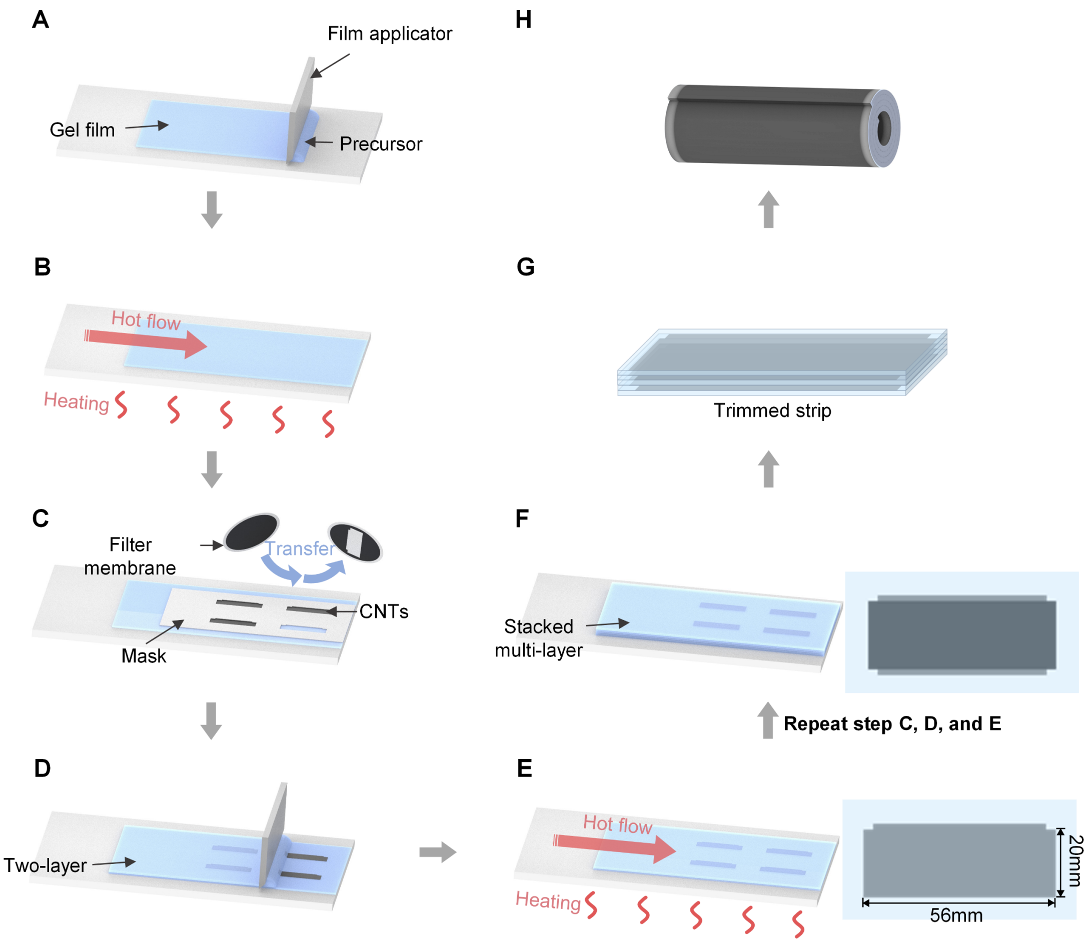

Figure S6. Fabrication process of cylindrical PVCg and PVCVAg actuators.

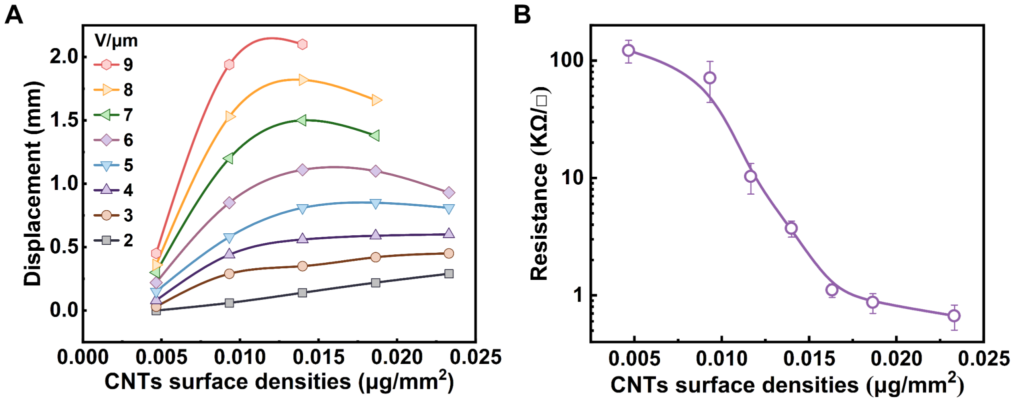

Figure S7. (A) Electroactive deformation of cylindrical PVCVAg actuator with different surface densities of CNT electrodes. (B) Resistance values of CNT electrodes with different surface densities.

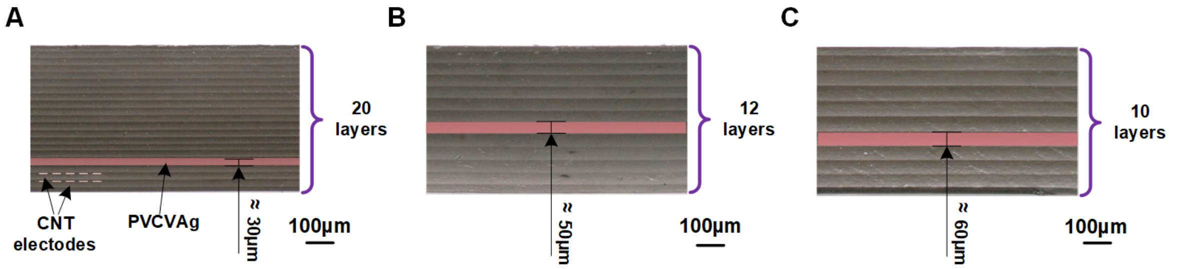

Figure S8. Cross-section view of multilayered PVCVAg actuators. (A) 20 layers with single-layer thickness of 30μm. (B) 12 layers with single-layer thickness of 50μm. (C) 10 layers with single-layer thickness of 60μm.

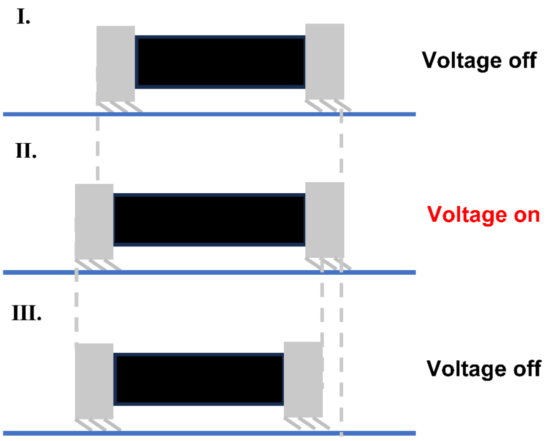

Figure S9. Mechanisms of motion for the insect-scale soft robot.

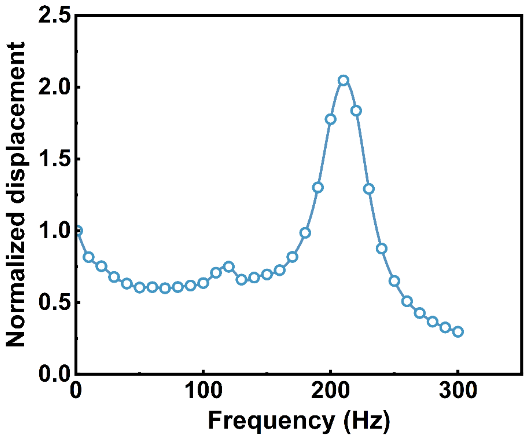

Figure S10. The electromechanical displacement of cylindrical PVCVAg actuator under continuously-varying frequencies. The displacement at 1 Hz is normalized to 1.

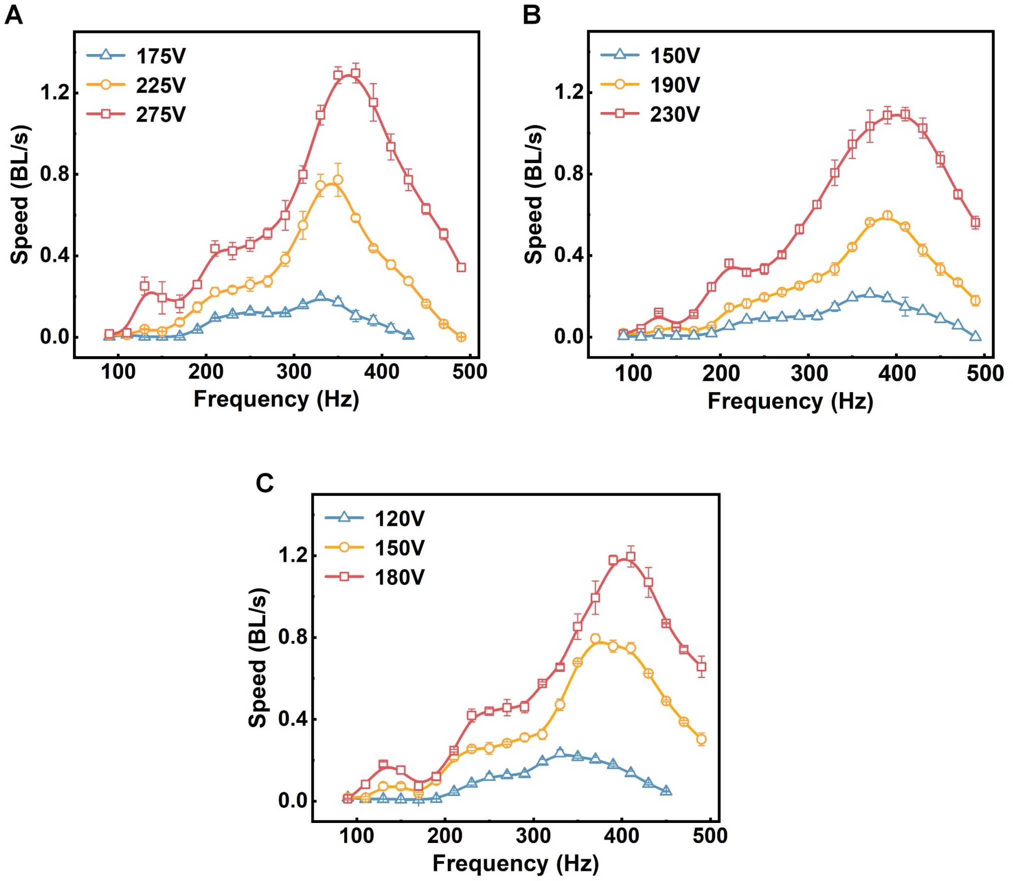

Figure S11. The crawling speeds of soft robot under different frequencies in which the cylindrical PVCVAg actuators are with different single-layer thicknesses. (A) 75 μm. (B) 60 μm. (C) 50 μm.

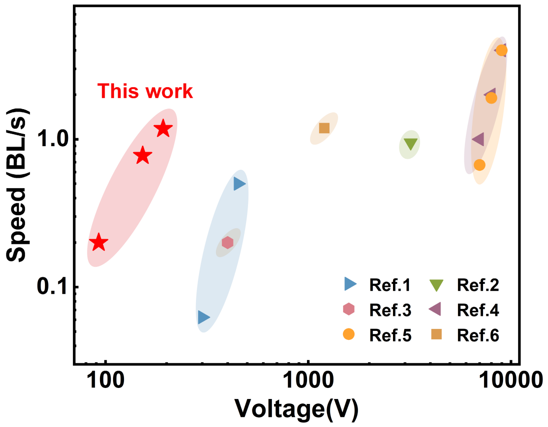

Figure S12. The relationship between crawling speed and driving voltage for EAP-based insect-scale soft robots with a body length smaller than 5 cm. The lowest driving voltage of our PVCVAg-based robots shows over 4× reduction versus state-of-the-art systems (e.g., 300V in Ref. 1).

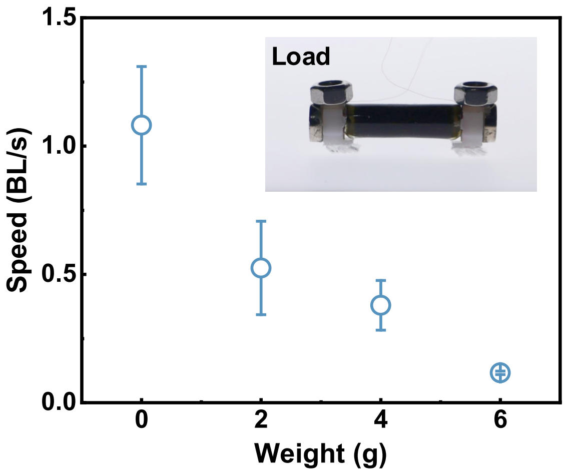

Figure S13. The relationship between the crawling speeds of the soft robot and the weight of the carried load. The inset shows the snapshot of the soft robot under a load of 4g. Error bars, standard deviation of the mean for n = 3 samples.

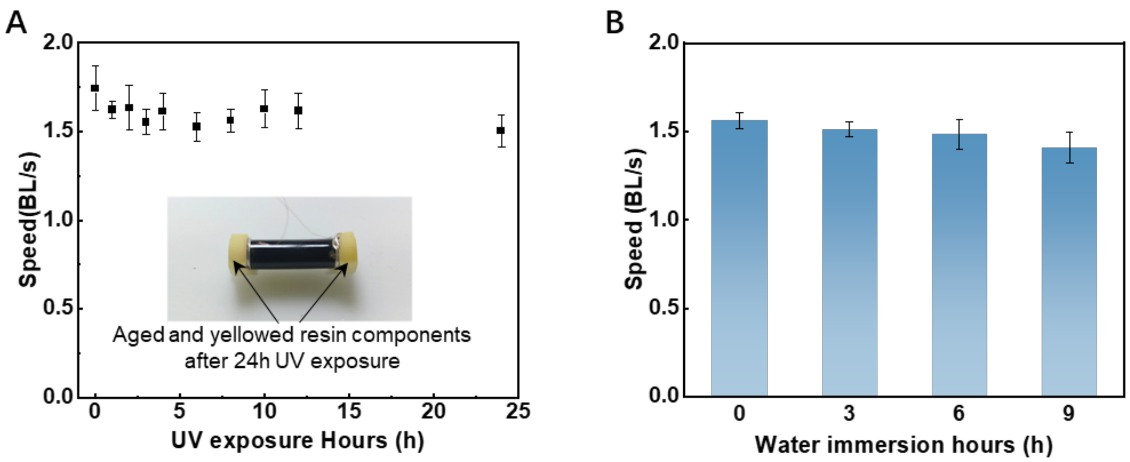

Figure S14. Environmental durability tests. (A) The relationship between the speed and UV exposure hours of robot. The inset shows a photograph of the robot after 24 hours of UV exposure. (B) The relationship between the speed and water immersion hours of robot. Error bars, standard deviation of the mean for n = 3 samples.

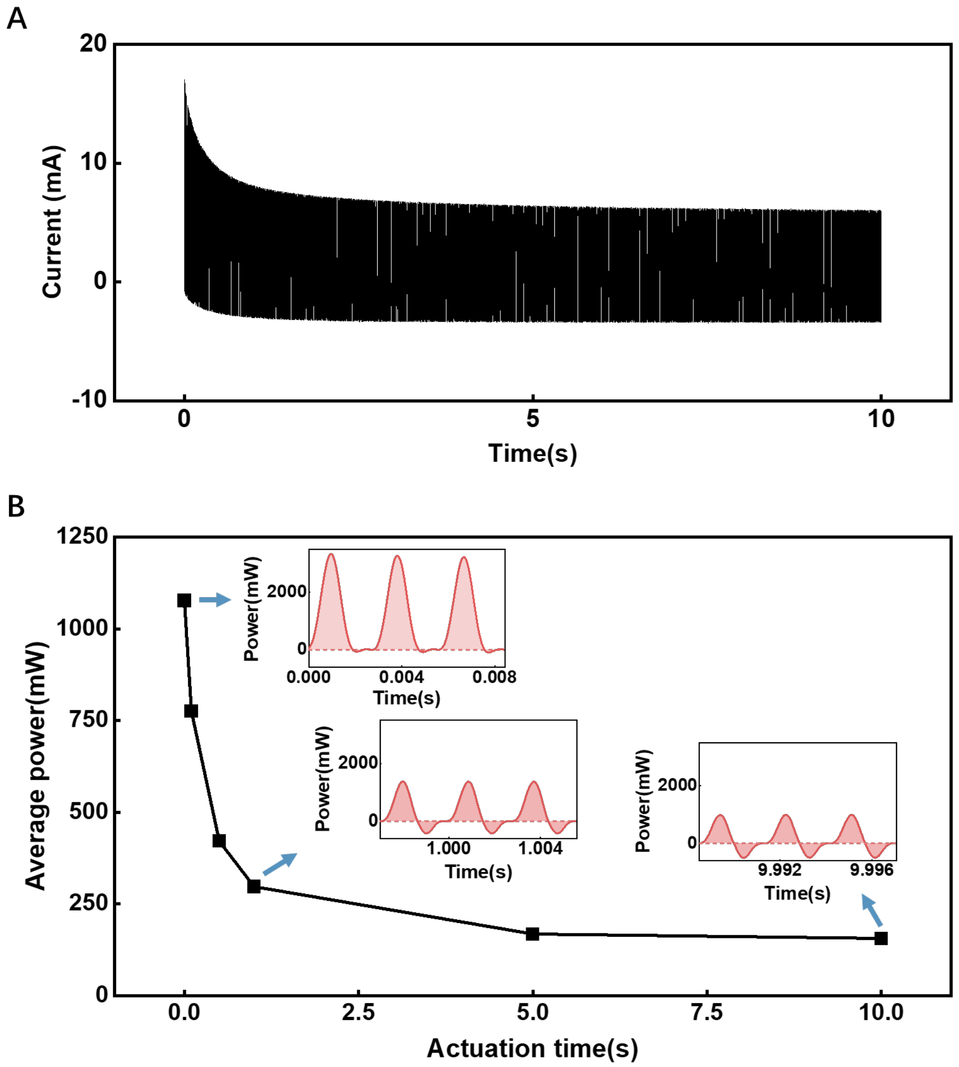

Figure S15. (A) The relationship between the current and actuation time of robot. (B) The relationship between the power consumption and actuation time of robot, and the instantaneous power consumption (the inset figures).

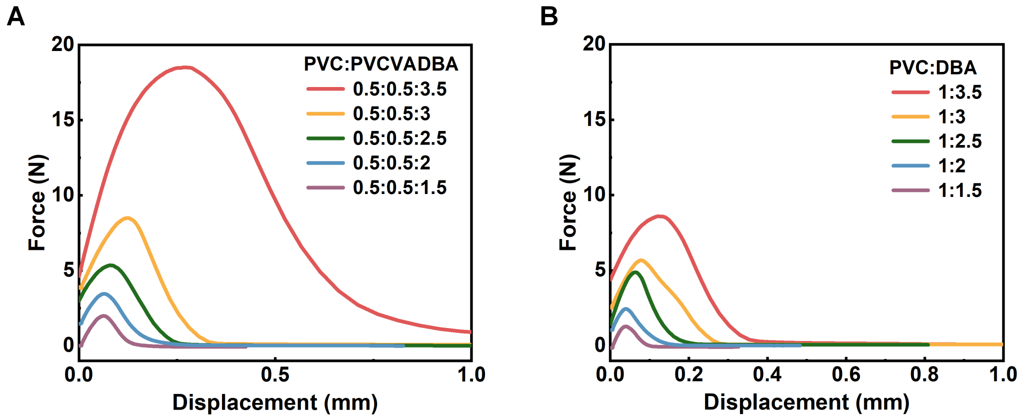

Figure S16. The relationship between adhesion force and displacement of PVCVAg and PVCg tested under an electric field of 2 V/μm. The adhesive area is 625mm^2.

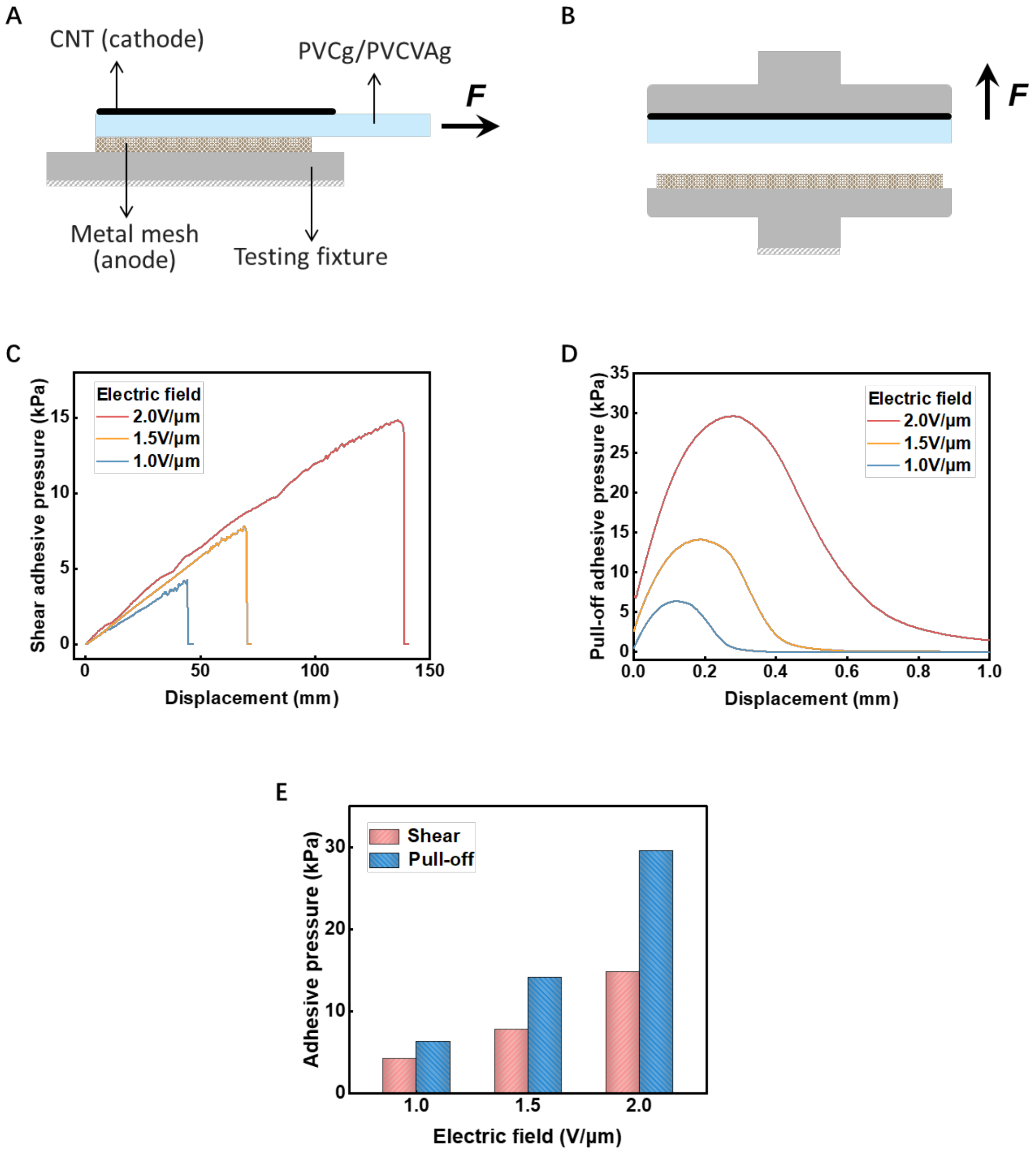

Figure S17. (A) Schematics of the shear test setup. (B) Schematics of the pull-off test setup. (C) The relationship between shear adhesive pressure and displacement of PVCVAg under different electric field. (D) The relationship between pull-off adhesive pressure and displacement of PVCVAg under different electric field. (E) Comparison of shear and pull-off adhesive pressures.

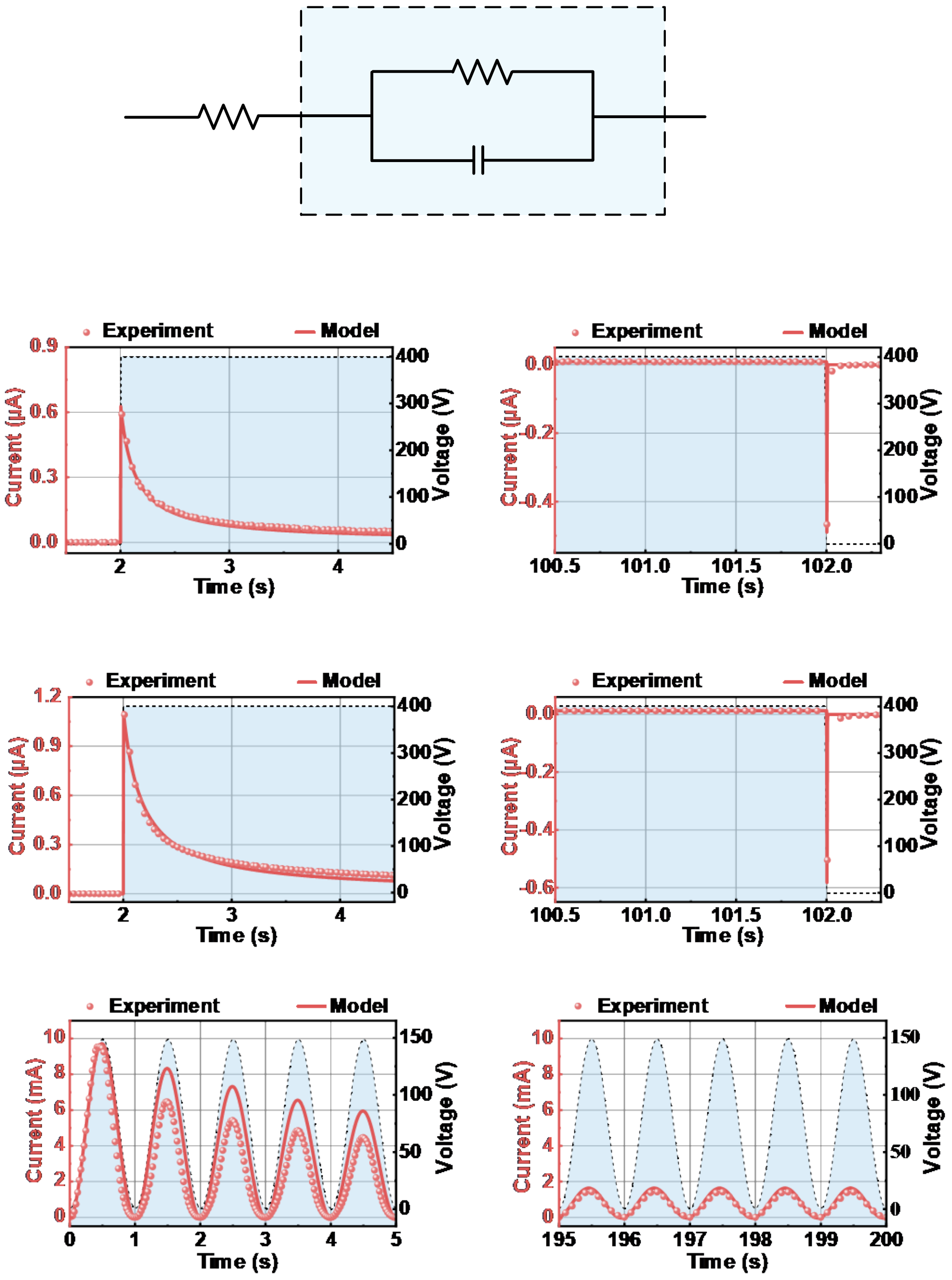

Figure S18. (A) The equivalent circuit model of PVCg/PVCVAg. (B) Comparison of the current response between simulation and experiment (Figure 4D) for PVCg electrostatic adhesion structures during electro-adhesion. (C) Comparison of the current response between simulation and experiment (Figure 4D) for PVCg-based electrostatic adhesion structures during electro-adhesion. (D) Comparison of the current response between simulation and experiment (Figure 6A) of rolled PVCVAg actuator.

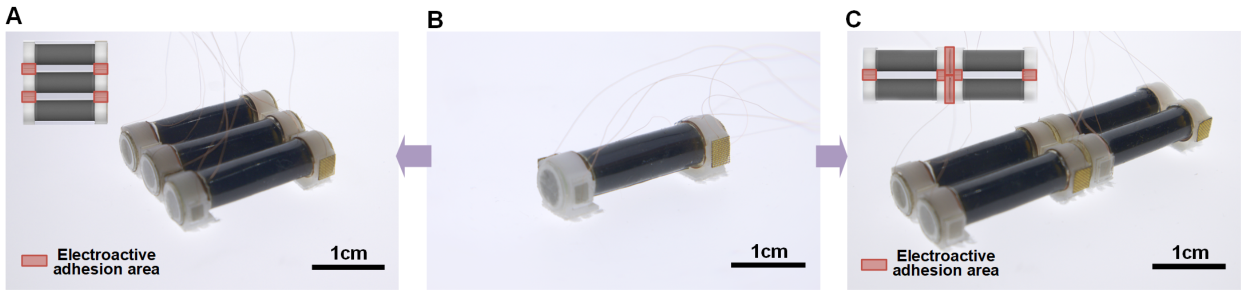

Figure S19. Images of insect-scale soft robots performing self-reconfiguration. (A) Self-reconfiguration in the parallel. (B) Single module. (C) Self-reconfiguration in the longitudinal directions.

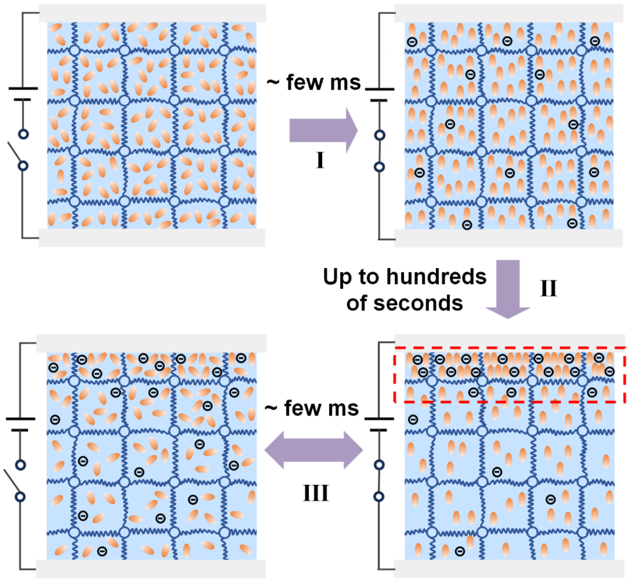

Figure S20. Schematic illustration of the evolution of the solvent-rich (S-R) layer within an electric field. Stage I. Orientation polarization of plasticizers under the applied electric field; Stage II. Migration of plasticizers and negative charges toward the anode establishes a S-R layer (red dashed box); Stage III. Stabilized electrical response is achieved after the formation of S-R layer.

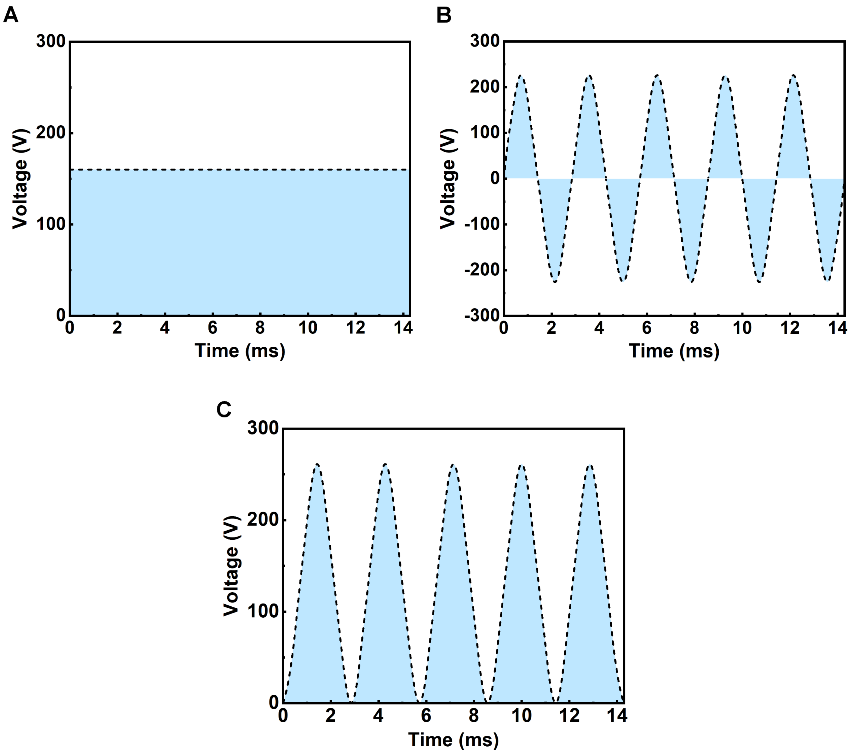

Figure S21. Waveforms of applied voltage in Figure. 6D. (A) DC 160 V, (B) AC -226/226 V and (C) AC 0/261 V.

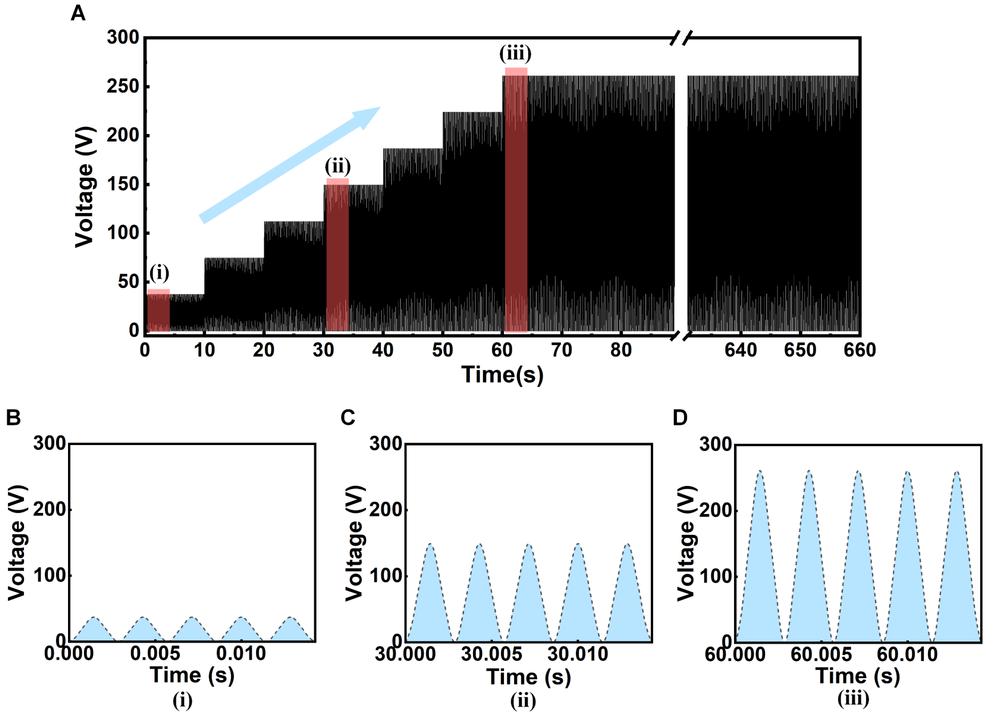

Figure S22. Waveforms of applied voltage for "pre-charging" in Figure. 6D. (A). The applied voltage with a period of 600s. (B) The local magnification of part (i). (C) The local magnification of part (ii). (D) The local magnification of part (iii).

Supplemental Tables

Robots	Size[cm]	Weight[g]	Connection mechanism	Independent mobility
Snail robot[7]	$\Phi 12$	1000	Permanent Magnetic + Suction	√
Granulobot[8]	$6.2\times\Phi 4.8$	98	Permanent Magnetic	√
CuBoat[9]	25×25	-	Permanent Magnetic	√
Eciton robotica[10, 11]	>23×3	120	Velcro	√
FreeSN[12]	7.7	480	Permanent Magnetic	×
Mori3[13]	18×18×1.5	240	Mechanical	√
StarBlocks[14]	7.2×7.2×7.2	50	Permanent Magnetic	√
FireAntV3[15]	15.9×6.5	-	Solder	×
Kubits[16]	2.5×2.5×2.5	17	Electro-Magnetic	×
FreeBOT[17]	12×12×12	308	Permanent Magnetic	√
Trimobot[18]	16×146	405	Mechanical	√
SMORES-EP[19, 20]	8×8×8	500	Electro-Magnetic	√
This Work	$3\times\Phi 1$	<1.2	Electrostatic	√

Table S1. Comparison of the self-reconfigurable robots between this work and the existing ones.

Component	Quantity	Weight[g]
Actuator	1	~0.875
End cap	2	0.220
Friction anisotropic feet	2	0.032
Electro-adhesion structure (PVCVAg, Circle)	1	0.012
Electro-adhesion structure (PVCVAg, Square)	2	0.010
Electro-adhesion structure (metal mesh, Circle)	1	0.021
Electro-adhesion structure (metal mesh, Square)	2	0.016
Total		~1.186

Table S2. The weight of components in the robot

Supplemental reference

[1] Ji, X., Liu, X., Cacucciolo, V., et al., An autonomous untethered fast soft robotic insect driven by low-voltage dielectric elastomer actuators, 2019, Sci. Robot., 4, eaaz6451.

[2] Li, W. B., Zhang, W. M., Gao, Q. H., et al., Electrically activated soft robots: speed up by rolling, 2021, Soft Robot., 8, 611.

[3] Feng, W., Sun, L., Jin, Z., Chen, L., Liu, Y., Xu, H., Wang, C., A large-strain and ultrahigh energy density dielectric elastomer for fast moving soft robot, 2024, Nat. Commun., 15, 4222.

[4] Li, T., Zou, Z., Mao, G., et al., Agile and resilient insect-scale robot, 2019, Soft Robot., 6, 133.

[5] Zhu, Y., Liu, N., Chen, Z., et al., 3D-printed high-frequency dielectric elastomer actuator toward insect-scale ultrafast soft robot, 2023, ACS Mater. Lett., 5, 704.

[6] Tang, C., Du, B., Jiang, S., Shao, Q., Dong, X., Liu, X.-J., Zhao, H., A pipeline inspection robot for navigating tubular environments in the sub-centimeter scale, 2022, Sci. Robot., 7, eabm8597.

[7] Zhao, D., Luo, H., Tu, Y., Meng, C., Lam, T. L., Snail-inspired robotic swarms: a hybrid connector drives collective adaptation in unstructured outdoor environments, 2024, Nat. Commun., 15, 3647.

[8] Saintyves, B., Spenko, M., Jaeger, H. M., A self-organizing robotic aggregate using solid and liquid-like collective states, 2024, Sci. Robot., 9, eadh4130.

[9] Zhang, L., Huang, Y., Cao, Z., Jiao, Y., Qian, H., Parallel self-assembly for a multi-USV system on water surface with obstacles, 2024, IEEE Trans. Autom. Sci. Eng., 1.

[10] Malley, M., Haghighat, B., Houel, L., Nagpal, R. 2020, 2020 IEEE International Conference on Robotics and Automation (ICRA), 4565.

[11] Malley, M., Rubenstein, M., Nagpal, R. 2017, 2017 IEEE/RSJ International Conference on Intelligent Robots and Systems (IROS), 6533.

[12] Tu, Y., Liang, G., Lam, T. L. 2022, 2022 International Conference on Robotics and Automation (ICRA), 4239.

[13] Belke, C. H., Holdcroft, K., Sigrist, A., Paik, J., Morphological flexibility in robotic systems through physical polygon meshing, 2023, Nat. Mach. Intell., 5, 669.

[14] Zhao, L., Wu, Y., Yan, W., et al., Starblocks: Soft actuated self-connecting blocks for building deformable lattice structures, 2023, IEEE Robot. Autom. Lett. , 8, 4521.

[15] Swissler, P., Rubenstein, M., FireAntV3: A modular self-reconfigurable robot towards free-form self-assembly using attach-anywhere continuous docks, 2023, IEEE Robot. Autom. Lett., 8, 4911

[16] Hauser, S., Mutlu, M., Ijspeert, A. J., Kubits: Solid-state self-reconfiguration with programmable magnets, 2020, IEEE Robot. Autom. Lett., 5, 6443.

[17] Liang, G., Luo, H., Li, M., Qian, H., Lam, T. L. 2020, 2020 IEEE/RSJ International Conference on Intelligent Robots and Systems (IROS), 6506.

[18] Zhang, Y., Song, G., Liu, S., Qiao, G., Zhang, J., Sun, H., A modular self-reconfigurable robot with enhanced locomotion performances: design, modeling, simulations, and experiments, 2016, J. Intell. Robot. Syst., 81, 377.

[19] Daudelin, J., Jing, G., Tosun, T., Yim, M., Kress-Gazit, H., Campbell, M., An integrated system for perception-driven autonomy with modular robots, 2018, Sci. Robot., 3, eaat4983.

[20] Tosun, T., Davey, J., Liu, C., Yim, M. 2016, 2016 IEEE/RSJ International Conference on Intelligent Robots and Systems (IROS), 45.
